# The geographical distribution and prevalence of *Echinococcus multilocularis* in animals in the European Union and adjacent countries: a systematic review and meta-analysis

**DOI:** 10.1186/s13071-016-1746-4

**Published:** 2016-09-28

**Authors:** Antti Oksanen, Mar Siles-Lucas, Jacek Karamon, Alessia Possenti, Franz J. Conraths, Thomas Romig, Patrick Wysocki, Alice Mannocci, Daniele Mipatrini, Giuseppe La Torre, Belgees Boufana, Adriano Casulli

**Affiliations:** 1 Finnish Food Safety Authority Evira (FINPAR), Elektroniikkatie 3, FI-90590 Oulu, Finland; 2 Department of Parasitic Zoonoses, IRNASA, CSIC, Cordel de Merinas, 40-52, 37008 Salamanca, Spain; 3 Department of Parasitology, National Veterinary Research Institute, 57 Partyzantów Avenue, 24-100, Puławy, Poland; 4 Department of Infectious, Parasitic and Immunomediated Diseases, Istituto Superiore di Sanitá, Viale Regina Elena, 299, 00161 Rome, Italy; 5 Friedrich-Loeffler-Institut, Federal Research Institute for Animal Health, Institute of Epidemiology, Südufer 10, 17493 Greifswald-Insel Riems, Germany; 6 Universität Hohenheim, FG Parasitologie 220 B, 70599 Stuttgart, Germany; 7 Dipartimento di Sanità Pubblica e Malattie Infettive, Sapienza University of Roma, Piazzale Aldo Moro 5, 00185 Rome, Italy; 8 European Reference Laboratory for Parasites (EURLP), Rome, Italy; 9 World Health Organization Collaborating Centre for the Epidemiology, Detection and Control of Cystic and Alveolar echinococcosis (in humans and animals), Rome, Italy

**Keywords:** *Echinococcus multilocularis*, Europe, Systematic review, Geographical distribution, Prevalence

## Abstract

**Background:**

This study aimed to provide a systematic review on the geographical distribution of *Echinococcus multilocularis* in definitive and intermediate hosts in the European Union (EU) and adjacent countries (AC). The relative importance of the different host species in the life-cycle of this parasite was highlighted and gaps in our knowledge regarding these hosts were identified.

**Methods:**

Six databases were searched for primary research studies published from 1900 to 2015. From a total of 2,805 identified scientific papers, 244 publications were used for meta-analyses.

**Results:**

Studies in 21 countries reported the presence of *E. multilocularis* in red foxes, with the following pooled prevalence (PP): low (≤ 1 %; Denmark, Slovenia and Sweden); medium (> 1 % to < 10 %; Austria, Belgium, Croatia, Hungary, Italy, the Netherlands, Romania and the Ukraine); and high (> 10 %; Czech Republic, Estonia, France, Germany, Latvia, Lithuania, Poland, Slovakia, Liechtenstein and Switzerland). Studies from Finland, Ireland, the United Kingdom and Norway reported the absence of *E. multilocularis* in red foxes. However, *E. multilocularis* was detected in Arctic foxes from the Arctic Archipelago of Svalbard in Norway.

**Conclusions:**

Raccoon dogs (PP 2.2 %), golden jackals (PP 4.7 %) and wolves (PP 1.4 %) showed a higher *E. multilocularis* PP than dogs (PP 0.3 %) and cats (PP 0.5 %). High *E. multilocularis* PP in raccoon dogs and golden jackals correlated with high PP in foxes. For intermediate hosts (IHs), muskrats (PP 4.2 %) and arvicolids (PP 6.0 %) showed similar *E. multilocularis* PP as sylvatic definitive hosts (DHs), excluding foxes. Nutrias (PP 1.0 %) and murids (PP 1.1 %) could play a role in the life-cycle of *E. multilocularis* in areas with medium to high PP in red foxes. In areas with low PP in foxes, no other DH was found infected with *E. multilocularis*. When fox *E. multilocularis* PP was >3 %, raccoon dogs and golden jackals could play a similar role as foxes. In areas with high *E. multilocularis* fox PP, the wolf emerged as a potentially important DH. Dogs and cats could be irrelevant in the life-cycle of the parasite in Europe, although dogs could be important for parasite introduction into non-endemic areas. Muskrats and arvicolids are important IHs. Swine, insectivores, murids and nutrias seem to play a minor or no role in the life-cycle of the parasite within the EU and ACs.

**Electronic supplementary material:**

The online version of this article (doi:10.1186/s13071-016-1746-4) contains supplementary material, which is available to authorized users.

## Background

Human alveolar echinococcosis (AE), caused by the metacestode stage of the tapeworm *Echinococcus multilocularis* is considered as one of the most pathogenic zoonosis in temperate and arctic regions of Europe [1]. The life-cycle of *E. multilocularis* involves small rodent intermediate hosts such as arvicolids and wild or domestic canid definitive hosts such as the red fox (*Vulpes vulpes*), the Arctic fox (*Vulpes lagopus*), the raccoon dog (*Nyctereutes procyonoides*) or the dog (*Canis lupus* f. *familiaris*). Humans can act as aberrant intermediate hosts and are infected through the ingestion of eggs excreted in the faeces of definitive hosts. Such faecal-oral infection can be acquired by contact with definitive hosts or through contamination of soil, food or possibly water [2]. In humans, the metacestode stage resembles a malignant neoplasia as it proliferates indefinitely by exogenous budding and slowly invades the surrounding tissue to produce tumour-like lesions [3]. Human alveolar echinococcosis is characterized by an asymptomatic incubation period of around 5–15 years [4].

In Europe, the human risk of *E. multilocularis* infection was considered in the past to be restricted to certain geographical regions. In fact, until the 1990s, only a ‘core’ area consisting of Eastern France, southern Germany, parts of Switzerland and Austria were known to be endemic for the disease [5]. More recently, the expansion of the parasite into several new areas such as the Baltic regions, Denmark, the Netherlands, Poland, Romania, Slovakia, Slovenia and the increase of human AE incidence in ‘core’ areas such as Austria, France and Switzerland, suggested that the disease was spreading in Europe and the incidence of human AE increasing at least in some regions [6–10]. Although greater awareness and the use of advanced diagnostic tools may have contributed to an improvement in the detection of *E. multilocularis* infection in animals and humans, epidemiological research conducted over the past 20 years, suggested the expansion of this parasite in European countries [9]. Factors such as change in landscape composition and use, vegetation, climate change, presence of good intermediate hosts, urbanization of foxes, changing human behavioural attitudes toward foxes, wildlife reintroduction, *E. multilocularis* host population dynamics as well as globalization have all been proposed as potential factors influencing the increase of *E. multilocularis* infection risk for Europe [9, 11, 12].

In the light of these concerns, the European Commission (EC) adopted a Commission Delegated Regulation (EU) No. 1152/2011 (14 July 2011). This was considered as a preventive health measure to control *E. multilocularis* infection in dogs and decrease the potential risk of AE infection in humans, in order to ensure continuous protection of Finland, Ireland, Malta and the United Kingdom (UK), countries that have remained free from *E. multilocularis* [13]. Regulation 1152/2011 described the obligations of these four European Union (EU) member states in implementing a pathogen surveillance programme for the detection of *E. multilocularis* in accordance with specific requirements regarding sampling, detection and reporting procedures [14]. It also stipulated that the EC had to review this regulation by December 2016 to assess the justification of these preventive health measures, in the light of scientific developments regarding *E. multilocularis* infection in animals. In response to Article 29 of Regulation (EC) No. 178/2002, in addition to an EC request, the European Food Safety Authority (EFSA) was tasked with assessing *E. multilocularis* infection in animals within the EU and neighbouring Adjacent Countries (ACs) (Albania, Belarus, Bosnia and Herzegovina, Iceland, Kosovo, Liechtenstein, Macedonia, Moldova, Montenegro, Norway, Russia, Serbia, Switzerland, Turkey and the Ukraine). To fulfil this requirement, EFSA funded a project to provide a comprehensive and quantitative assessment of the current knowledge on *E. multilocularis* using a systematic review (SR) approach (GP/EFSA/AHAW/2012/01: *Echinococcus multilocularis* infection in animals).

The current SR provides an overview of the distribution and prevalence of *E. multilocularis* in the EU and ACs derived from both scientific and grey literature. In addition, the purpose of this review was to systematically determine the geographical distribution of *E. multilocularis* and the known wild and domestic definitive and intermediate hosts. The retrieved information was used to compile tables on the occurrence of *E. multilocularis* or highlight the lack of reliable reports. When available, data on *E. multilocularis* prevalence and worm burden of definitive hosts was reported. The importance of the various definitive and intermediate host species in the life-cycle of *E. multilocularis* in different parts of the EU and ACs was assessed and gaps in our knowledge were identified.

## Methods

### Bibliographic searches

This SR and meta-analysis followed the Cochrane and PRISMA Group guidelines [15] and the systematic search was carried out using the Documentation Service for literature search at the Istituto Superiore di Sanità, Rome, Italy. The STN International-Fiz Karlsruhe platform [16] was used for database searching carried out on the 5^th^ November 2013 and again on the 11^th^ February 2015 in order to identify articles that had been published since the initial search. The results of these two searches were then combined. Searches were carried out using the Medical Literature Analysis and Retrieval System Online (MEDLINE), Excerpta Medica Database (EMBASE), Science Citation Index (SciSearch), Biological Abstracts (BIOSIS), Centre for Agricultural Bioscience International (CABI) and Google Scholar. Databases were searched using keywords associated with the Boolean operators “AND” and “OR”. The question mark (“?”) was used to expand searches by looking for words with similar prefixes using more than one letter (i.e. “echinococc?” was used to search for “echinococcus”, “echinococci”, “echinococcosis” and “echinococcoses”). The hashtag (“#”) was used to expand searches by looking for words with similar prefixes using one letter (i.e. dog# was used to search for “dog” or “dogs”).

Different combinations of words and Boolean operators were used in order to narrow results retrieved and maximise the number of relevant studies returned. The full electronic search strategy, including any limits used was: [*Echinococcus multilocularis* OR (*Echinococcus* AND *Multilocularis*) OR E# Multilocularis OR Alveolar Echinococcosis OR A# Echinococcosis] AND (Dog OR Dogs OR Cat OR Cats OR *Canis* OR *Felis* OR Canid? OR Felid? OR Wolf OR Wolves OR Animal OR Animals OR Fox OR Foxes OR *Vulpes* OR Ferret OR Ferrets OR Rodent OR Rodents OR Rodentia OR Nutria# OR Muskrat# OR Jackal# OR Arvicolid? OR Arvicolinae OR Worm Burden OR Host OR Hosts OR Hosted) AND (Occurrence# OR Geographic? Distribut? OR Geographic? Diffus? OR Incidence# OR Frequency OR Epidemic Outbreak# OR Endemic Outbreak# OR Prevalence# OR Epidemiology)]. If the title or abstract did not give a clear indication of relevance, the full text was screened. After this initial selection, full-text articles were evaluated for eligibility, in accordance with the inclusion/exclusion criteria described below. Data extraction was performed independently by two researchers and any disagreements were resolved either by consensus among researchers or through arbitration by an additional independent researcher. If database outcomes overlapped, all duplicated articles were removed. EU reports and conference proceedings were searched using the keywords “European Union report, “EU report”,”, “conference proceedings”, “*Echinococcus multilocularis*”, “*E. multilocularis*” and “alveolar echinococcosis”. Unpublished epidemiological data on *E. multilocularis* available within individual member states was collected from the National Reference Laboratories for Parasites in Europe [17] using a questionnaire (Additional file 1: Text S1). Searches for Bachelor, Masters and PhD theses were carried out using the keywords “*Echinococcus multilocularis*” and “alveolar echinococcosis”. A list of databases used for retrieving theses is available in Additional file 2: Text S2*.* Review Manager [18] software was used to prepare and maintain this SR.

### Study selection

Studies eligible for inclusion were defined a priori and fulfilled the following criteria: (i) studies published from 1900 to 2015; (ii) studies based on cross-sectional or cohort design; (iii) primary research studies either published or in press; (iv) reports on wild or domestic hosts of *E. multilocularis*; (v) studies published in English, German, French, Polish, Finnish, Dutch, Spanish or Italian.

**Fig. 1:**
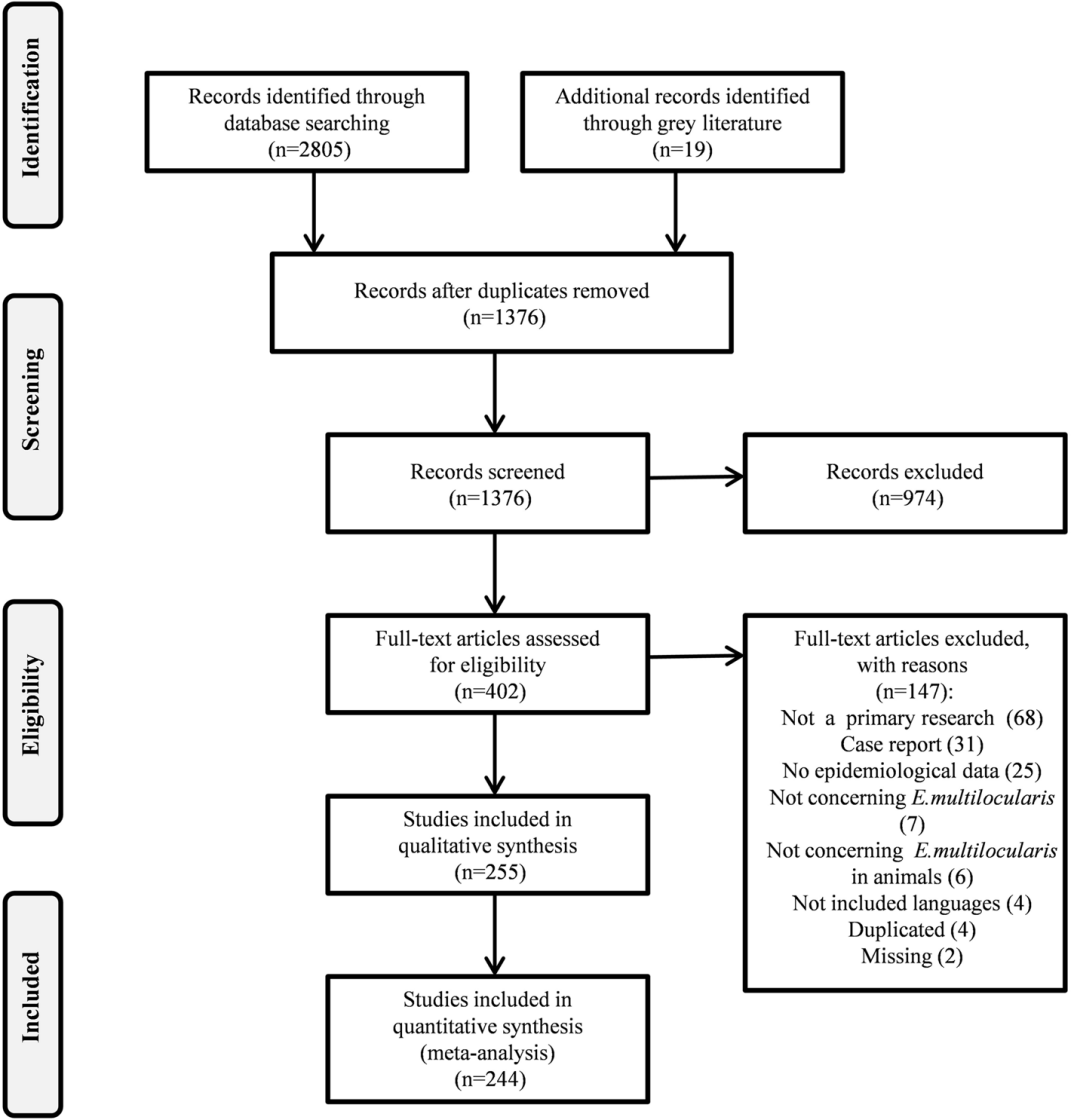
The flow chart represents the algorithm of record/article selection

The list of included articles is available in Additional file 3: Text S3. Studies providing data from outside Europe and ACs, case reports, reports on *E. multilocularis* in humans, studies on agents other than *E. multilocularis* (e.g. *Echinococcus granulosus*), reviews and letters or editorials without original data were all excluded from this SR. The list of excluded articles is available in Additional file 4: Text S4. The study selection process was carried out according to the PRISMA statement [15] and is reported using the flow chart shown in Fig. 1.

### Eligibility for inclusion in the meta-analyses

Studies included in the meta-analyses were those that reported prevalence data (total number of studied animals and number of positive animals) and studies with a definition of a geographical area (whenever possible the Nomenclature of Territorial Units for Statistics designated as NUTS level 1, 2 and 3 was used) [19]. When studies originated from different geographical areas or when they were conducted within the same geographical area but at different time intervals (e.g. during distinct years or months) they were divided into sub-studies. Data were extracted from each study independently. If the same samples were tested using different diagnostic methods, only data derived from the sedimentation and counting technique (SCT) or the intestinal scraping technique (IST) were included in the analysis. Studies reporting prevalence data obtained exclusively by enzyme-linked immunosorbent assays (ELISAs), designed to detect pathogen-specific copro-antigens in DHs, were subsequently excluded from the meta-analysis because of the low specificity of this test. When it was not possible to accurately assign the proportion of data reported per country, meta-analysis was not performed.

### Statistical approach and meta-analyses

Statistical analysis was conducted using the statistical software Stats Direct 2.8.0 (Stats Direct Ltd., Altrincham, UK). To perform the meta-analysis, animal species were divided into two main groups, definitive (DH) and intermediate (IH) hosts. The DHs included the red fox (*Vulpes vulpes*), the Arctic fox (*Vulpes lagopus*), the raccoon dog (*Nyctereutes procyonoides*), wild canids (wolf, *Canis lupus;* golden jackal, *Canis aureus*), the dog (*Canis lupus* f. *familiaris*) and the cat (*Felis silvestris* f. *catus*). The IHs included arvicolid rodents (including *Arvicola* spp., *Myodes* (syn. *Clethrionomys*) *glareolus* and *Microtus* spp. but excluding the muskrat *Ondatra zibethicus*), muskrat, nutria or coypu (*Myocastor coypus*), murid rodents (including *Apodemus* spp., *Micromys minutus, Mus musculus* and *Rattus* spp.), insectivores (including *Sorex* spp., *Neomys fodiens* and *Talpa europaea*) and swine (domestic *Sus scrofa* f. *domesticus* and the wild boar *Sus scrofa*). Each meta-analysis group included studies conducted in the same geographical area, at the European level, national level and using the three NUTS levels [19]. Meta-analyses were not stratified for the years/months in which the studies were conducted.

Since all included studies were cross-sectional, meta-analyses on proportions were performed. The Cochran’s Q test was performed to assess the degree of heterogeneity between studies, and the I^2^ index was used to describe the percentage of total variation across studies as a result of heterogeneity. If the *p*-value from the Cochran’s Q test was < 0.05 and the I^2^ statistic was > 50 %, heterogeneity was found and a random-effect model was applied. However, if heterogeneity was not detected, a fixed-effect model was used. A forest plot was produced to describe the pooled analysis; this showed the single prevalence of the studies and the pooled proportion with relative 95 % confidence intervals (CIs). Publication bias was quantified by inspection of funnel plots and computation of Begg and Egger’s probability values [20, 21].

### Quality assessment

The quality of all included studies was assessed independently by two researchers using the Newcastle-Ottawa Scale (NOS) according to the Cochrane Handbook for Systematic Reviews [22, 23]. The NOS was modified for use on an animal model. Quality assessment could not be performed on grey literature.

## Results and discussion

Bibliographic searches identified 2,805 scientific papers, of which 1,429 were deleted due to duplications. At the end of the search, 1,376 papers were identified of which 974 were excluded based only on title and abstract screening. A total of 402 full-text papers were assessed for eligibility, data were extracted from 255 studies and it was possible to perform meta-analyses on 244 studies (Fig. 1). The quality assessment carried out using the modified NOS, allowed the allocation of a maximum 7-star rating to any one individual study. A score of 5 or 6 was given to 108, 9, 1, 17 and 8 studies on foxes, raccoon dogs, wild canids, dogs and cats, respectively. A lower score (4 or 3) was assigned to 79 studies on foxes, 7 on raccoon dogs, 2 on wild canids, 9 on dogs and 12 on cats. A similar scoring for intermediate hosts showed that 2 studies on muskrats and 5 on arvicolids had a 5 or 6 rating. Four or three star ratings were assigned to 6 studies on muskrats, 11 on arvicolids, 4 on murids, 2 on nutria, 1 on insectivores and 1 on swine, respectively.

### Geographical distribution and prevalence of *Echinococcus multilocularis*

#### Red foxes

Data regarding the geographical distribution and prevalence of *E. multilocularis* in red foxes were extracted from reports published for the period between 1968 and 2014 (Table 1).

**Table 1:** Pooled prevalence of *Echinococcus multilocularis* in red foxes

Country	No. of studies included	Pooled prevalence (%)	95 % CI (%)	Time range of studies (years)	Reference
Austria	13	8.0	2.0–17.0	1989–2000	[37–43]
		6.5	4.3–9.1	2000–2005	[44]
Belgium	17	13.5	3.6–28.4	1993–2000	[45–49]
		8.0	3.0–16.0	2000–2012	[24, 49–55]
Croatia	3	2.3	1.1–15.6	2013–2016	[24; Relja Beck, personal communication]
Czech Republic	10	12.7	6.1–21.2	1994–1999	[78–83]
		16.0	4.0–35.0	2005–2010	[30, 31, 40]
Denmark	6	0.5	0.2–0.8	2000–2013	[24, 25, 27, 39]
Estonia	4	24.5	13.0–38.2	2003–2014	[84–86]
Finland	8	0	0	2000–2013	[32, 45, 199]
France	72	23	16.0–30.0	1968–2000	[87–96]
		13.9	9.8–18.6	2000–2010	[30, 31, 40, 97–104]
Germany	303	13.8	12.3–15.3	1973–2000	[39, 41, 105–143]
		29.2	26.0–32.4	2000–2012	[24, 30, 31, 40, 109, 117, 131, 144–151]
Hungary	42	8.0	5.6–10.7	2008–2013	[24, 56–59]
Ireland	9	0	0	2003–2013	[31, 199–201]
Italy	26	0.55^a^	na	1997–2000	[60]
		1.5	0.5–2.9	2000–2012	[24, 60–66]
Latvia	14	36.8	22.2–52.9	2002–2008	[152]
Lithuania	2	58.0	54.0–62.0	2001–2006	[153, 154]
Luxembourg	9	16.7	9.4–25.6	2005–2012	[24, 30, 31, 40]
Netherlands	14	4.0	2.0–6.0	1995–2000	[67–69]
		4.7	1.9–9.0	2000–2013	[30, 31, 40, 49, 54, 67–72]
Poland	69	2.0	1.3–3.0	1994–2000	[155–160]
		14.8	9.6–20.8	2000–2014	[161–171]
Romania	32	0	0	1981–1992	[73]
		4.5	2.9–6.4	2000–2010	[74, 75]
Slovakia	3	23	12.3–15.3	1998–1999	[260]
	64	27.3	24.4–30.3	2000–2013	[24, 28, 31, 40, 164, 172–184]
Slovenia	2	0.9	0.2–5.3	2002–2005	[28, 29]
Spain	1	0^a^	na	2012	[24]
Sweden	10	0.2	0.1–0.3	2000–2012	[24, 30–36]
United Kingdom	8	0	0	2000–2014	[24, 160, 199, 200, 202]
Liechtenstein	1	34.9^a^	na	1990–1992	[70]
Norway	29	0	0	2000–2014	[24, 31, 32, 203–206]
Switzerland	59	26.8	23.0–30.7	1988–2000	[39, 185–191]
		17.0	6.1–31.9	2000–2003	[24, 30, 40, 192–198]
Ukraine	4	2.8	0.1–9.0	2000–2010	[76, 77]

^(a)^ Prevalence estimate from only one study, not pooled prevalence

*Abbreviations*: na, not applicable

**Fig. 2:**
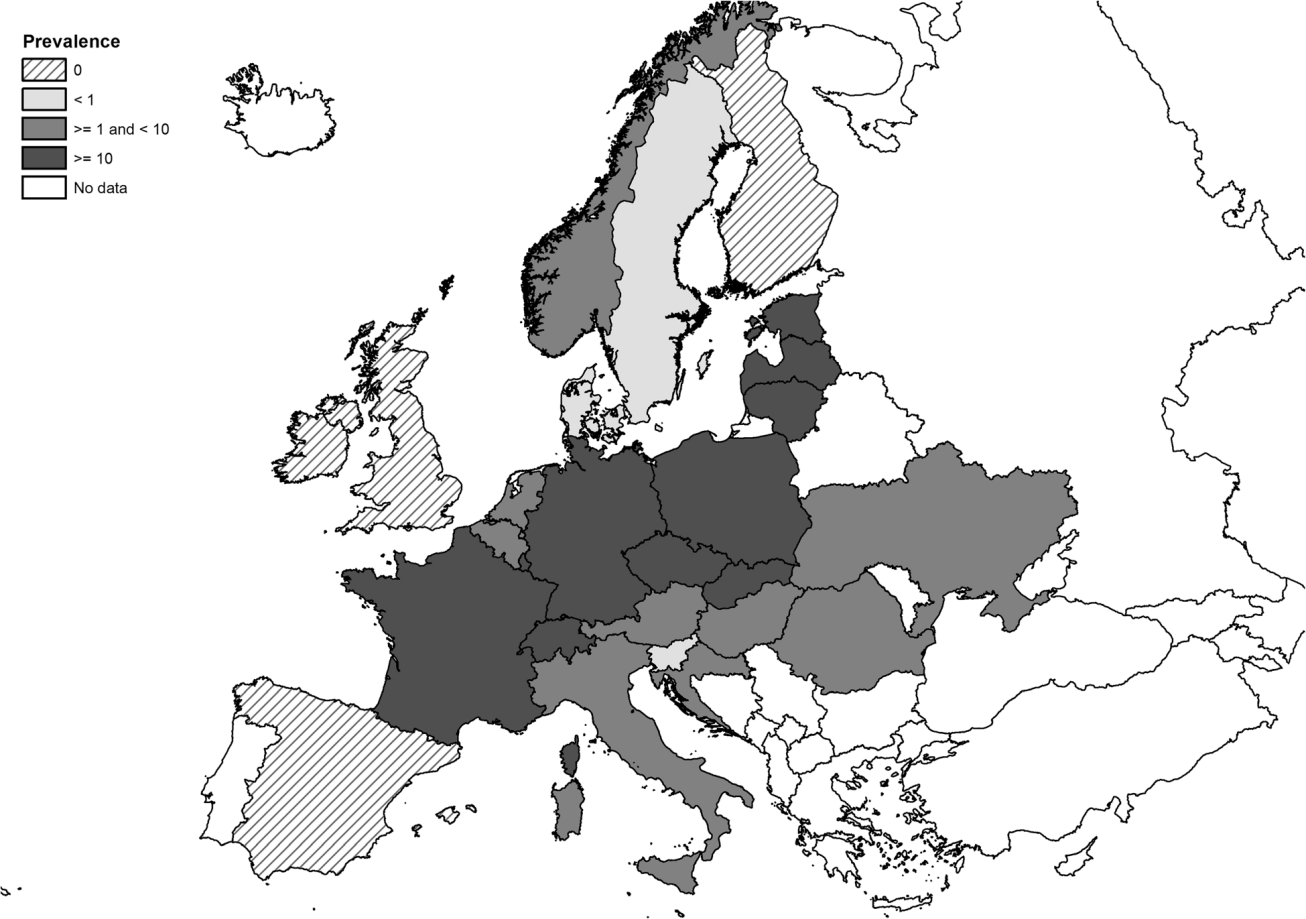
Pooled prevalence of *Echinococcus multilocularis* in red and Arctic foxes within the European Union and adjacent countries at national level (data obtained from studies performed after 2000). Note: the pooled prevalence data for Norway originated only from Arctic foxes on the Svalbard islands; prevalence data from Spain originated from single studies

**Fig. 3:**
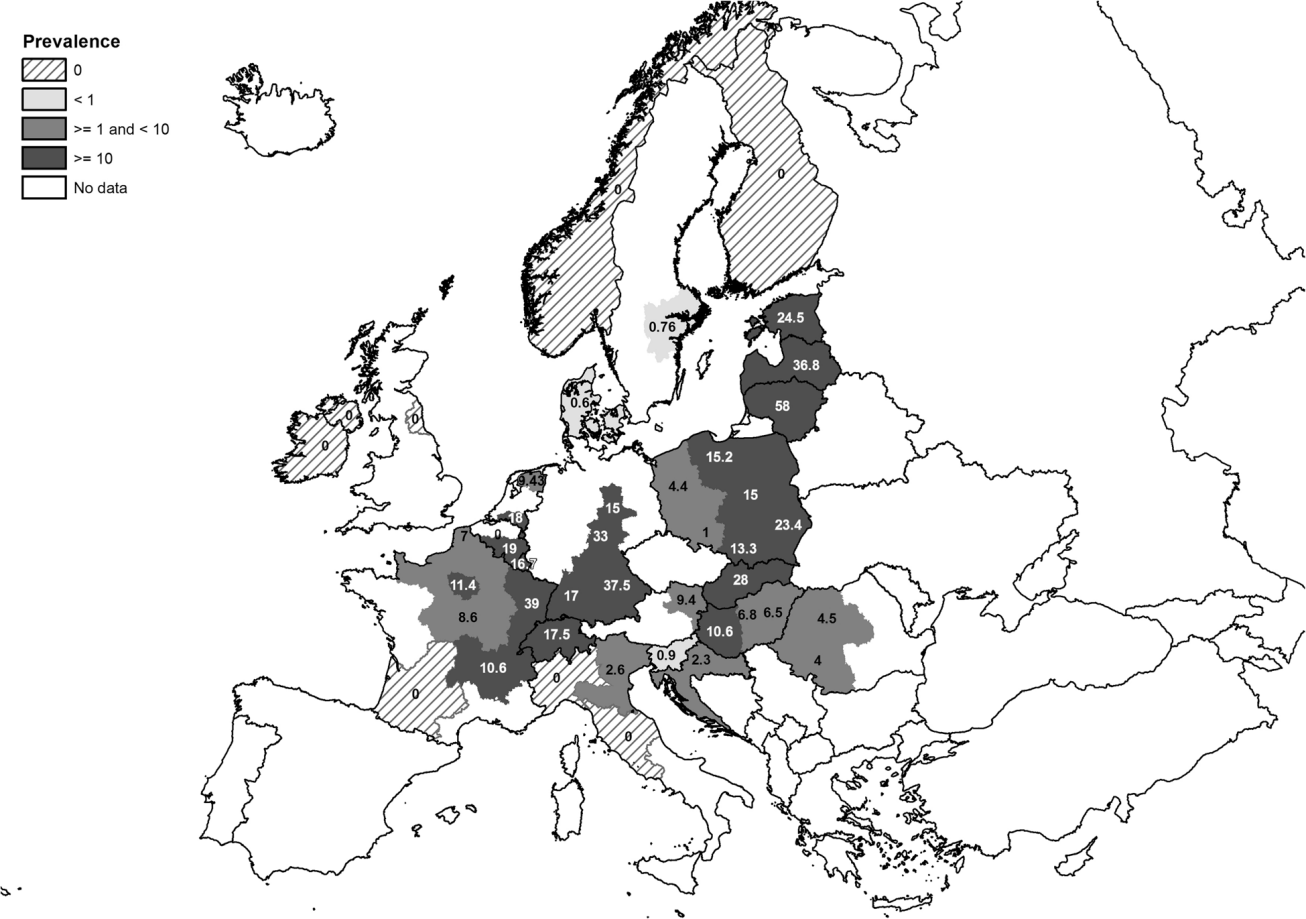
Pooled prevalence of *Echinococcus multilocularis* in red foxes within the European Union and adjacent countries at NUTS 1 level (data obtained from studies after 2000). Note: prevalence data from the Netherlands and Sweden originated from single studies; only studies reporting NUTS information were taken into account

A total of 192 papers describing the distribution and prevalence of *E. multilocularis* in foxes were used in the meta-analyses. A preliminary ranking of *E. multilocularis* infection in red foxes based on pooled prevalence allowed us to identify three main groups (Table 1). A low prevalence group included countries with a pooled prevalence of ≤1 %, namely Denmark [24–27], Slovenia [28, 29] and Sweden [24, 30–36]; a medium prevalence group with a pooled prevalence of > 1 % but ≤ 10 %, which included Austria [37–44], Belgium [24, 45–55], Croatia [24, Relja Beck, personal communication], Hungary [24, 56–59], Italy [24, 60–66], the Netherlands [30, 31, 40, 49, 54, 67–72], Romania [73–75] and the Ukraine [76, 77], whereas the high prevalence territories had a pooled prevalence of > 10 % and included the Czech Republic [30, 31, 40, 78–83], Estonia [84–86, L. Laurimaa, personal communication], France [30, 31, 40, 87–104], Germany [24, 30, 31, 39–41, 105–151], Latvia [152], Lithuania [153, 154], Poland [155–171], Slovakia [24, 28, 31, 40, 164, 172–184], Liechtenstein [70] and Switzerland [24, 30, 39, 40, 185–198]. The occurrence and pooled prevalence of *E. multilocularis* in foxes in the EU and ACs is shown in Fig. 2. The highest prevalence estimates for *E. multilocularis* in red foxes seem to be concentrated in central and north-eastern Europe. A more detailed map of the geographical distribution and pooled prevalence of *E. multilocularis* in red foxes at a NUTS 1 level is shown in Fig. 3. Studies from four countries, namely Finland, Ireland, the UK and Norway, reported the absence of *E. multilocularis* in red foxes [24, 31, 32, 158, 196–203]. *Echinococcus multilocularis* in Arctic foxes in Norway was documented only for the Arctic Archipelago of Svalbard [207, 208].

### Other definitive hosts

Five potential DHs of *E. multilocularis* other than red foxes were identified in the screened literature; four wild animal species, the Arctic fox [21, 24, 209], the raccoon dog [24–26, 32, 77, 152, 154, 168, 169, 175, 177, 199, 210–213], the golden jackal [24, 214] and the wolf [31, 77, 215] and two domestic animal species, dogs [24, 30, 72, 80, 95, 101, 122, 142, 168, 169, 177, 193, 195, 199, 200, 216–225] and cats [24, 37, 66, 80, 113, 115, 121, 122, 132, 133, 142, 168, 169, 177, 216, 219, 223, 226–230]. The geographical distribution and prevalence of *E. multilocularis* for these DHs are summarized in Table 2.

**Table 2:** Pooled prevalence of *Echinococcus multilocularis* in Arctic foxes, raccoon dogs, wild canids (golden jackal and wolf), cats and dogs

Country	Host	No. of studies included	Pooled prevalence (%)	95 % CI (%)	Time range of studies (years)	Reference
Austria	Cat	1	0^a^	na	2004–2005	[216]
	Dog	1	0^a^	na	2004–2005	[216]
Cyprus	Dog	1	0^a^	na	2012	[24]
Czech Republic	Cat	2	50	8.0–92.0	1997–2004	[80, 230]
	Dog	1	1.8^a^	na	1998	[80]
Denmark	Raccoon dog	4	0	0	2011–2013	[24–26]
	Cat	1	0.6^a^	na	2004–2005	[216]
	Dog	1	0^a^	na	2004–2005	[216]
	Wild canids	1	0^a^	na	2012	[24]
Estonia	Raccoon dog	1	1.6^a^	na	2012	[210]
Finland	Raccoon dog	4	0	0	2012	[24, 199, 200]
	Dog	1	0^a^	na	2012	[24]
	Wild canids	2	0	0	2013	[24]
France	Cat	3	1.5	0.2–7.9	1989–2012	[24, 216, 226]
	Dog	6	0.4	0.1–0.9	1988–2013	[30, 95, 101, 216–218]
Germany	Raccoon dog	4	2.5	0.1–7.9	1998–2008	[211–213]
	Cat	14	0.6	0.3–1.0	1973–2005	[24, 113, 115, 121, 122, 132, 133, 142, 216, 219, 227, 228]
	Dog	6	0.3	0.2–0.3	1973–2012	[24, 122, 142, 216, 219]
Hungary	Wild canids	2	4.7	0.1–15.3	2007–2013	[24, 214]
Italy	Cat	1	0^a^	na	2004–2005	[216]
	Dog	2	0	0	2004–2012	[24, 216]
Latvia	Raccoon dog	1	21^a^	na	2002–2008	[152]
	Wild canids	1	5.9^a^	na	2003–2008	[215]
Lithuania	Raccoon dog	1	8.2^a^	na	2001–2006	[154]
	Dog	1	0.8^a^	na	2005–2006	[220]
Luxembourg	Cat	1	0^a^	na	2004–2005	[216]
	Dog	1	0^a^	na	2004–2005	[216]
Malta	Dog	2	0	0	2012–2013	[199, 200]
Netherlands	Raccoon dog	1	0^a^	na	2012	[24]
	Dog	2	0	0	2004–2013	[72, 216]
	Cat	1	0.3^a^	na	2004–2005	[216]
Poland	Raccoon dog	3	10.4	4.1–19.3	nr	[24, 168, 169]
	Cat	2	0	0	nr	[168, 169]
	Dog	2	0	0	nr	[168, 169]
Slovakia	Raccoon dog	3	28.0	4.0–64.0	2002–2007	[24, 175, 177]
	Cat	2	0	0	2002–2012	[24, 177]
	Dog	5	0.4	0.1–1.3	2002–2012	[24, 177, 221, 222]
	Wild canids	1	0^a^	na	2013	[24]
Sweden	Raccoon dog	1	0^a^	na	2000–2009	[32]
	Dog	2	0	0	2012–2013	[24]
	Wild canids	2	0	0	2012–2013	[24]
United Kingdom	Cat	1	0^a^	na	2004–2005	[216]
	Dog	1	0^a^	na	2004–2005	[216]
Norway	Arctic fox	2	5.8	3.9–8.2	1996–2004	[21, 24, 209]
Switzerland	Cat	2	4.6	0.3–13.6	1999–2012	[24, 223]
	Dog	6	1.2	0.1–3.4	1996–2013	[24, 193, 195, 223–225]
Ukraine	Raccoon dog	1	0^a^	na	1998–2010	[77]
	Wild canids	1	0^a^	na	1998–2010	[77]

^a^Prevalence estimate from only one study, not pooled prevalence

*Abbreviations*: na, not applicable; nr, not reported

Pooled prevalence results showed that sylvatic animals, excluding red foxes, are more frequently infected than domestic species. The two species showing high *E. multilocularis* prevalence were the raccoon dog and the golden jackal. In general, high *E. multilocularis* prevalence in these two species correlated with high infection rates in foxes. Importantly, the raccoon dog is currently not established in some areas that are deemed free of *E. multilocularis* (e.g. Ireland, Malta and the UK), but is present in high numbers in Finland. A third species, with high prevalence rates was the Arctic fox [21, 24, 209], which is only present in a few northern countries, namely northern Russia, Iceland, and the Norwegian Arctic Archipelago of Svalbard, in addition to a small population on the Scandinavian peninsula.

Dogs and cats do not seem to be important in terms of prevalence and are found to be infected only in some areas of high *E. multilocularis* pooled prevalence in red foxes such as Czech Republic [80, 212], Germany [24, 113, 115, 121, 122, 132, 133, 142, 216, 219, 227, 228], France [24, 30, 95, 101, 216–218, 226] and Switzerland [24, 193, 195, 223–225]. However, dogs can be regarded as potentially relevant hosts considering *E. multilocularis* introduction into areas that are free of the parasite by travelling from endemic to distant (non-endemic) areas with their owners, and also with regard to transmission in endemic areas because of their closer association with humans than sylvatic DHs.

Information on *E. multilocularis* worm burden in definitive hosts from EU countries and ACs was only available in a few studies for red foxes (43/190) [25–27, 29, 34, 38, 55–59, 61, 63, 68, 75–77, 79, 80, 85, 99, 102, 110, 119, 127, 140, 152–154, 161, 165–167, 173–176, 179–181, 189, 191, 192], raccoon dogs (3/17) [32, 154, 213], dogs (1/23) [224] and cats (5/19) [80, 113, 132, 169, 228]. In contrast, no data were available on *E. multilocularis* worm burden of wild canids and Arctic foxes for the same regions.

#### Intermediate hosts

Potential IHs of *E. multilocularis* screened in this study included the muskrat [51, 113, 121, 136, 231–241], arvicolids [24, 30, 32, 39, 51, 79, 93, 95, 101, 121, 141, 187, 189–191, 207, 209, 223, 224, 242–257], murids [51, 79, 95, 101, 168, 169, 224, 229, 242, 246, 250, 251, 253, 256], nutria [231, 233], swine [32, 73, 220, 258] and insectivores [24, 79, 101, 251] (Table 3). For the majority of countries, the distribution of the prevalence of *E. multilocularis* in muskrats and arvicolids matched that (although the prevalence was lower) in red foxes and was similar to the pooled prevalence of *E. multilocularis* in other sylvatic DHs (Table 2). Muskrats and arvicolids are thus potentially good sentinels to investigate the presence of *E. multilocularis* in specific settings.

**Table 3:** Pooled prevalence of *Echinococcus multilocularis* in arvicolids, muskrat, nutria, swine (domestic and wild), insectivores and murids

Country	Host	No. of studies included	Pooled prevalence (%)	95 % CI (%)	Time range of studies (years)	Reference
Belgium	Arvicolids	4	0.2	0.0–0.6	2003–2004	[24]
	Muskrat	2	16.0	7.0–28.0	2003–2006	[24, 232]
	Insectivores	1	0^a^	na	2003–2004	[24]
	Murids	1	0^a^	na	2003–2004	[24]
Czech Republic	Arvicolids	4	1.3	0.1–3.7	1997	[79]
	Insectivores	4	0	0	1997	[79]
	Murids	4	0	0	1997	[79]
Finland	Arvicolids	3	0	0	2000–2012	[24, 32]
	Swine	2	0	0	2000–2009	[32]
France	Arvicolids	36	4.8	1.6–9.7	1975–1995	[93, 95, 101, 242–249, 255, 257]
	Murids	5	0.97	0.7–1.3	1979–1985	[95, 101, 242, 246, 256]
	Muskrat	2	1.1	0.2–2.8	1985–2010	[233, 234]
	Nutria	1	5.8^a^	na	2002–2003	[233]
	Insectivores	1	0^a^	na	1999–2000	[101]
Germany	Arvicolids	6	0.6	0.4–1.0	1979–1995	[121, 141, 250]
	Muskrat	51	3.8	2.8–4.9	1974–2003	[113, 121, 136, 231, 235–237, 240–241]
	Nutria	1	0.4^a^	na	2010	[231]
	Murids	1	0^a^	na	1979–1986	[250]
	Swine	1	5.3^a^	na	2004	[259]
Lithuania	Swine	1	0.4^a^	na	2005–2006	[220]
Luxembourg	Muskrat	1	1.8^a^	na	nr	[238]
Netherlands	Muskrat	1	0.06^a^	na	1998–1999	[239]
Poland	Arvicolids	6	0	0	2004–2006	[251]
	Insectivores	4	0	0	2004–2006	[251]
	Murids	9	0	0	nr	[168, 169, 251]
Romania	Arvicolids	1	1.4^a^	na	1989–2010	[262]
	Swine	2	0	0	1989	[73]
Sweden	Swine	6	0^a^	na	2000–2009	[32]
Norway	Arvicolids	5	27.0	18.0–37.0	1999–2009	[30, 39, 207, 209]
	Swine	1	0^a^	na	2000–2009	[32]
Switzerland	Arvicolids	26	13.3	10.8–16.1	1993–2008	[187, 189–191, 223, 224, 252–254]
	Murids	3	0	0	1999–2002	[224, 229, 253]
	Swine	1	10^a^	na	nr	[258]

^a^Prevalence estimate from only one study, not pooled prevalence

*Abbreviations*: na, not applicable, nr, not reported

Among murids, *Apodemus* spp. was the host with the highest *E. multilocularis* prevalence [24, 79, 224, 242, 246, 250, 251, 253, 256]. In France, *E. multilocularis* prevalence in these species was similar to that reported for *Microtus* spp*.* [256]. Only one study on *E. multilocularis* infection in *Mus musculus* in France is known to exist [95]. In general, murids have not frequently been found positive for *E. multilocularis* [24, 79, 101, 168, 169, 224, 229, 242, 246, 250, 251, 253]. However, the number of studies (*n* = 14) and the number of murids examined remains small (*n* = 2,610). None of the screened insectivores were positive for *E. multilocularis* [24, 79, 101, 251] but the number examined was small (*n* = 531). Although swine seem to play no role in the life-cycle of this parasite, *E. multilocularis* infections in swine were reported from Germany [259], Lithuania [220] and Switzerland [258] and therefore this animal species could potentially be regarded as a domestic IH sentinel (Table 3). Data regarding *E. multilocularis* in definitive and intermediate hosts in EU countries and ACs are summarised in Table 4.

**Table 4:** Data on *Echinococcus multilocularis* infection in definitive and intermediate hosts

Country	Definitive hosts (DHs)				Intermediate hosts (HIs)		
	Red fox	Raccoon dog	Other (sylvatic)	Other (domestic)	Arvicolids	Muskrat	Other
Austria^(L)^	Yes	na	na	No (cat, dog)	na^(S)^	na^(S)^	na
Belgium	Yes	na	na	na	Yes	Yes	No (murids; insectivores)
Bulgaria^(N)^	na^(S)^	na^(S)^	na	na	na^(S)^	na^(S)^	na
Croatia^(L)^	Yes	na^(S)^	na	na	na^(S)^	na^(S)^	na
Cyprus^(F)^	na	na	na^(S)^	No (dog)	na^(S)^	na	na^(S)^
Czech Republic	Yes	na	na	Yes (cat, dog)	Yes	na	No (murids; insectivores)
Denmark^(L)^	Yes	No	No (wild canids)	Yes (cat), no (dog)	na^(S)^	na^(S)^	na
Estonia^(L)^	Yes	Yes	na	na	na^(S)^	na^(S)^	na
Finland^(F)^	No	No	No (wild canids)	No (dog)	No	na	No (swine)
France	Yes	na	na	Yes (cat, dog)	Yes	Yes	Yes (nutria, murids); No (insectivores)
Germany	Yes	Yes	na	Yes (cat, dog)	Yes	Yes	Yes (nutria, swine); No (murids)
Greece^(F)^	na^(S)^	na^(S)^	na	na	na^(S)^	na^(S)^	na
Hungary^(L)^	Yes	na	Yes (golden jackal)	na	na^(S)^	na^(S)^	na
Ireland^(F)^	No	na^(S)^	na	na	na^(S)^	na^(S)^	na
Italy	Yes	na	na	No (cat, dog)	na^(S)^	na^(S)^	na
Latvia^(L)^	Yes	Yes	Yes (wild canids)	na	na^(S)^	na^(S)^	na
Lithuania	Yes	Yes	na	Yes (dog)	na^(S)^	na^(S)^	Yes (swine)
Luxembourg	Yes	na	na	No (cat, dog)	na	Yes	na
Malta^(F)^	na	na	na^(S)^	No (dog)	na^(S)^	na	na^(S)^
Netherlands	Yes	No	na	Yes (cat) No (dog)	na	Yes	na
Poland^(L)^	Yes	Yes	na	No (cat, dog)	No	na^(S)^	No (murids; insectivores)
Portugal^(N)^	na^(S)^	na^(S)^	na	na	na	na^(S)^	na
Romania	Yes	na	na	na	Yes	na	No (swine)
Slovakia^(L)^	Yes	Yes	No (wild canids)	Yes (dog) No (cat)	na^(S)^	na^(S)^	na
Slovenia^(L)^	Yes	na	na	na	na^(S)^	na^(S)^	na
Spain^(F)^	No	na^(S)^	na	na	na^(S)^	na^(S)^	na
Sweden	Yes	No	No (wild canids)	No (dog)	na	na^(S)^	No (swine)
United Kingdom^(F)^	No	na^(S)^	na	No (cat, dog)	na^(S)^	na^(S)^	na
Albania^(N)^	na^(S)^	na^(S)^	na	na	na	na	na
Belarus^(N)^	na^(S)^	na^(S)^	na	na	na	na	na
Bosnia and Herzegovina^(N)^	na^(S)^	na^(S)^	na	na	na^(S)^	na^(S)^	na
Macedonia^(N)^	na^(S)^	na^(S)^	na	na	na^(S)^	na^(S)^	na
Iceland^(N)^	na^(S)^	na^(S)^	na	na	na^(S)^	na^(S)^	na
Kosovo^(N)^	na^(S)^	na^(S)^	na	na	na^(S)^	na^(S)^	na
Liechtenstein^(L)^	Yes	na	na	na	na^(S)^	na^(S)^	na
Moldova^(N)^	na^(S)^	na^(S)^	na	na	na^(S)^	na^(S)^	na
Montenegro^(N)^	na^(S)^	na^(S)^	na	na	na^(S)^	na^(S)^	na
Norway	No	na^(S)^	Yes (Arctic fox)	na	Yes	na	No (swine)
Russia^(N)^	na^(S)^	na^(S)^	na	na	na^(S)^	na^(S)^	na
Serbia^(N)^	na^(S)^	na^(S)^	na	na	na^(S)^	na^(S)^	na
Switzerland	Yes	na	na	Yes (cat, dog)	Yes	na	No (murids) Yes (swine)
Turkey^(N)^	na^(S)^	na^(S)^	na	na	na^(S)^	na^(S)^	na
Ukraine^(L)^	Yes	No	No (wild canids)	na	na^(S)^	na^(S)^	na

Countries with ^(F)^are those potentially free from *Echinococcus multilocularis*. Countries with ^(N)^have no data on *Echinococcus multilocularis* in DHs or IHs. Countries with ^(L)^have detected the presence of the parasite, but data on the main DH and/or IH are lacking. Cells marked with ^(S)^indicate that those animal species should be screened, if present, either to ascertain the absence of the parasite or the presence of specific hosts important for maintaining the parasite life-cycle. When the main DH and IH are not present, alternative and suitable hosts to be screened should be found (e.g. in Malta and Cyprus)

*Abbreviation*: na, not applicable

### Ranking of hosts (other than red foxes) in the life-cycle of *Echinococcus multilocularis*

#### Definitive hosts

In order to clarify the importance of other screened DHs in the life-cycle of *E. multilocularis*, pooled prevalence for each DH, other than red foxes were generated (Table 5). The ranking of pooled prevalence in DHs could be used to hypothesise the importance of the different DHs in the life-cycle of *E. multilocularis*.

**Table 5:** Pooled prevalence of *Echinococcus multilocularis* in definitive hosts other than red foxes

Species (or group of species)	No. of studies included	Pooled prevalence (%)	95 % CI (%)	Time range of studies (years)	Location of the studies (Reference)
Dog (*Canis lupus* f. *familiaris*)	39	0.3	0.2–0.5	1973–2013	Slovakia [24, 177, 221, 222], Germany [24, 122, 142, 147, 216], Denmark [216], France [30, 95, 101, 216–218], Switzerland [24, 193, 195, 223–225], Czech Republic [79], Italy [24, 216], Austria [216], Luxembourg [216], The Netherlands [72, 216], Poland [168, 169], Lithuania [220], Cyprus [24], Finland [24], Malta [199, 200], Sweden [24], United Kingdom [216]
Cat (*Felis silvestris* f. *catus*)	31	0.5	0.3–0.8	1973–2013	Germany [24, 113, 135, 121, 122, 132, 133, 142, 216, 219, 227, 228], Switzerland [24, 223], Slovakia [24, 177], France [24, 216, 226], Czech Republic [79, 230], Austria [216], Luxembourg [216], The Netherlands [216], Italy [216], Poland [168, 169], Denmark [216], United Kingdom [216]
Arctic fox (*Vulpes lagopus*)	2	9.0	6.0–12.0	1996–2013	Norway, Svalbard only [21, 24, 209]
Raccoon dog (*Nyctereutes procyonoides*)	24	2.2	0.8–4.1	1998–2013	Lithuania [154], Latvia [152], Slovakia [24, 175, 177], Denmark [24–26], Germany [211–213], Ukraine [77], Sweden [32], Poland [24, 168, 169], Finland [24, 199, 200], Netherlands [31], Estonia [210]
Wolf (*Canis lupus*)	8	1.4	0.3–3.4	1998–2013	Latvia [215], Ukraine [77], Sweden [24], Slovakia [24], Denmark [24], Finland [24]
Golden jackal (*Canis aureus*)	2	4.7	0.1–15.3	2007–2013	Hungary [24, 214]

Ranking based on an *E. multilocularis* pooled prevalence of > 3 %, resulted in the following order (high to low rank): red fox, Arctic fox, golden jackal, raccoon dog and wolf. Although data on the golden jackal and the Arctic fox are scarce [21, 24, 209, 214], they provide evidence in support of these two animal species serving as potentially important DHs of *E. multilocularis.* Despite some uncertainties due to the low number of studies regarding these two species, data have nevertheless been included in this report for the following reasons: (i) these are the only data available for the golden jackal and the Arctic fox; and (ii) parasite prevalence in the studied individuals was high (Arctic fox, 9 %, 95 % CI: 6–12; golden jackal, 4.7 %, 95 % CI: 0.1–15.3), which is indicative of the potentially important role that these species could play in the maintenance and transmission of *E. multilocularis*. Interestingly, Arctic foxes are restricted to the northern area of the EU and ACs because of their habitat needs, but the golden jackal population seems to have an increasing trend of migrating from eastern EU countries and ACs towards the west, which should be taken into account when considering the potential future spread of *E. multilocularis*.

#### Intermediate hosts

In order to clarify the importance of the screened IHs in the life-cycle of *E. multilocularis*, the pooled prevalence for each IH group was determined (Table 6). Pooled prevalence in the screened IH groups showed that muskrats and arvicolids (muskrats, *n* = 25,985; arvicolids, *n* = 65,956) (and more specifically *Arvicola* spp.) are important in the life-cycle of *E. multilocularis.* For nutria (*n* = 650) and murids (*n* = 2,610), the number of animals screened was too low to draw any robust conclusions, although it seems that they could play a role in the life-cycle of *E. multilocularis* in areas with a sustained medium to high pooled prevalence in red foxes [24, 79, 95, 101, 168, 169, 224, 229, 231, 233, 242, 246, 250, 251, 253, 256]. Swine and insectivores seem to play no role in the life-cycle of *E. multilocularis* within the EU and ACs.

**Table 6:** Pooled prevalence of *Echinococcus multilocularis* in intermediate hosts

Species (or group of species)	No. of studies included	Pooled prevalence (%)	95 % CI (%)	Time range of studies (years)	Location of the studies (Reference)
Muskrat (*Ondatra zibethicus*)	57	4.2	3.0–5.6	1974–2010	Belgium [51, 232], Germany [113, 121, 136, 231, 235–237, 240, 241], France [233, 234], The Netherlands [239], Luxembourg [238]
Nutria (*Myocastor coypus*)	2	1.04	0.41–1.96	2002–2010	Germany [231], France [233]
Arvicolids (*Arvicola* spp., *Myodes glareolus*, *Microtus* spp.)	91	6.0	4.0–8.2	1979–2013	Belgium [51], Germany [121, 141, 250], Switzerland [187, 189-191, 223, 224, 252–254], France [93, 95, 101, 242–249, 255–257], Czech Republic [79], Poland [251], Romania [262], Finland [24, 32], Norway [30, 39, 207, 209]
Murids (*Mus musculus*, *Rattus rattus*, *Rattus norvegicus*, *Apodemus* spp., *Micromys minutus*)	23	1.1	0.2–2.8	1979–2009	Belgium [51], Germany [250], Switzerland [224, 229, 253], France [95, 101, 242, 246, 256], Czech Republic [79], Poland [168, 169, 251]
Swine (*Sus scrofa* f. *domesticus*) and wild boar (*Sus scrofa*)	14	0.001	0–0.006	1989–2009	Romania [73], Germany [259], Lithuania [220], Sweden [32], Finland [32], Norway [32], Switzerland [258]
Insectivores (*Sorex* spp., *Talpa europaea*, *Neomys fodiens*)	10	0	0	1997–2006	Belgium [51], France [101], Czech Republic [79], Poland [251]

### The importance of different definitive hosts in countries classified as having low, medium and high prevalence rates of *Echinococcus multilocularis*

#### Definitive hosts

Considering that the number of studies and the number of animals screened in many cases were too low for drawing robust conclusions, the following comments should be regarded as tentative.

The importance of each screened DH, according to country, was stratified by the pooled prevalence of *E. multilocularis* in red foxes (or Arctic foxes in Svalbard, Norway). The resulting classification, with regard to *E. multilocularis* infection, enabled us to group countries into zero, low, medium or high prevalence regions (Table 7). The raccoon dog [24–26, 32, 199, 200], the wolf [31], the dog [31, 216] and cat [216] were screened in countries with low (including absence of the parasite) *E. multilocularis* prevalence in foxes. None of these DHs, at this level of fox prevalence, seem to sustain the life-cycle of *E. multilocularis*, although issues relating to the representativeness of the sample number should be taken into account since, occasionally, the number of screened animals was low (raccoon dogs, *n* = 3,833; dogs, *n* = 27,638; cats, *n* = 13,498).

**Table 7:** Grouping of countries according to *Echinococcus multilocularis* prevalence in red foxes in relation to definitive (DH) and intermediate (IH) hosts

Level of prevalence in red foxes (%)	Country	DHs	Pooled prevalence (%)	IHs	Pooled prevalence (%)
0 (0)	Finland	Raccoon dog	0	Arvicolids	0
		Dog	0^a^		
		Wild canids (Wolf)	0	Swine	0
	Ireland	No data	No data	No data	No data
	United Kingdom	Dog	0^a^	No data	No data
		Cat	0^a^		
0< >1 (low)	Denmark	Cat	0.60^a^	No data	No data
		Dog	0^a^		
		Raccoon dog	0		
		Wild canids (Wolf)	0^a^		
	Sweden	Raccoon dog	0^a^	Swine	0^a^
		Dog	0^a^		
		Wild canids (Wolf)	0^a^		
	Slovenia	No data	No data	No data	No data
1< >10 (medium)	Austria	Dog	0^a^	No data	No data
		Cat	0^a^		
	Belgium	No data	No data	Muskrat	16.00
				Insectivores	0^a^
				Arvicolids	0.20
				Murids	0^a^
	Croatia	No data	No data	No data	No data
	Hungary	Wild canids (Golden jackal)	4.70	No data	No data
	Italy	Dog	0	No data	No data
		Cat	0^a^		
	Netherlands	Cat	0.30^a^	Muskrat	0.06^a^
		Dog	0^a^		
		Raccoon dog	0^a^		
	*Norway (5.82 %)* ^b^ *(only Svalbard archipelago)*	No data	No data	Arvicolids	27.50
				Swine	0^a^
	Romania	No data	No data	Arvicolids	1.40^a^
				Swine	0^a^
	Ukraine	Wild canids (Wolf)	0^a^	No data	No data
		Raccoon dog	0^a^		
>10 (high)	Poland	Raccoon dog	10.40	Insectivores	0
		Dog	0	Arvicolids	0
		Cat	0	Murids	0
	Switzerland	Dog	1.20	Arvicolids	13.30
		Cat	4.60	Murids	0
				Swine	10^a^
	Czech Republic	Dog	1.80^a^	Insectivores	0
		Cat	50.00	Arvicolids	1.30
				Murids	0
	Germany	Dog	0.30	Muskrat	3.80
				Nutria	0.40^a^
		Cat	0.60	Arvicolids	0.60
		Raccoon dog	2.50	Murids	0
				Swine	5.30^a^
	Estonia	Raccoon dog	1.60^a^	No data	No data
	France	Dog	0.40	Muskrat	1.10
				Nutria	5.80^a^
		Cat	1.50	Insectivores	0^a^
				Arvicolids	4.80
				Murids	0.97
	Liechtenstein	No data	No data	No data	No data
	Lithuania	Dog	0.80^a^	Swine	0.40^a^
		Raccoon dog	8.20^a^		
	Latvia	Raccoon dog	21.00^a^	No data	No data
		Wild canids (Wolf)	5.90^a^		
	Slovakia	Dog	0.40	No data	No data
		Cat	0		
		Raccoon dog	28.00		
		Wild canids (Wolf)	0^a^		
No data	Luxembourg	Dog	0^a^	Muskrat	1.80^a^
		Cat	0^a^		

^a^Prevalence estimate from only one study, not pooled prevalence

^b^Arctic foxes sampled

For countries stratified in the medium *E. multilocularis* prevalence group, golden jackals [24, 214], if present, seem to participate in the life-cycle of the parasite, with prevalence estimates roughly similar to those reported for red foxes in the same countries [56–59, 120]. By contrast, wolves [77], dogs [24, 72, 216] and cats [216] seem to play no role in countries with medium *E. multilocularis* prevalence levels in foxes  [24, 30, 31, 37–44, 49, 54, 60–72]. For countries with high *E. multilocularis* prevalence levels, raccoon dogs [24, 152, 154, 168, 169, 175, 177, 210–213] are also important in the life-cycle of the parasite, with prevalence estimates of between one-seventh and two-thirds of the pooled prevalence in foxes. An exception is evident in Slovakia, where the pooled *E. multilocularis* prevalence in foxes [24, 31, 40, 164, 172–184, 260] was similar to the prevalence found in raccoon dogs (~27 %) [24, 175, 177]. Importantly, in countries with a high prevalence, an additional DH (i.e. wolf) seems to join the life-cycle of *E. multilocularis*, although with a lower prevalence (one-sixth) than that reported for foxes and raccoon dogs [215].

With regard to domestic hosts (dogs and cats), only a very low prevalence of *E. multilocularis* could be found and only in highly endemic situations (Table 7), and thus these hosts seem to be of minor importance in the life-cycle of the parasite in Europe and ACs, especially when a zero, low or medium *E. multilocularis* prevalence is found in foxes. In addition, cats have been shown to be unsuitable hosts for *E. multilocularis*, because full maturity of the parasite is often not attained in the feline intestine [261].

#### Intermediate hosts

In countries with a low (including 0) *E. multilocularis* pooled prevalence in foxes, only two types of IHs have been screened, namely arvicolids (in Finland) [24] and swine (in Finland and Sweden) [32] whereas in other countries such as Ireland, Slovenia and the UK no IHs have been inspected for the prevalence of *E. multilocularis.* Therefore, to interpret these results, the potential importance of those IHs in medium- and high-prevalence situations should first be assessed. Muskrats and arvicolids seem to be the only IHs for *E. multilocularis* in medium-prevalence rated countries. In muskrats, a pooled prevalence of 16 % was recorded in Belgium [51, 232] and a prevalence of 0.06 % in the Netherlands [236] where the pooled prevalence for *E. multilocularis* in foxes was 8 and 4.7 %, respectively. Similarly, in Norway (Arctic fox pooled prevalence 5.8 %), Romania (fox pooled prevalence 4.5 %) and Belgium (fox pooled prevalence 8 %), the pooled prevalence for *E. multilocularis* in arvicolids was 27.5 % [30, 39, 207, 209], 1.4 % [262] and 0.2 % [51], respectively. In countries with a high *E. multilocularis* prevalence, the prevalence estimates were high for arvicolids (13.3 %) [187, 189–191, 223, 224, 252–254] and pigs (10 %) [258] in Switzerland (fox pooled prevalence 17 %), muskrats (3.8 %) [113, 121, 136, 231, 235–237, 240, 241] and pigs (5.3 %) [259] in Germany (fox pooled prevalence 29.2 %) and arvicolids (4.8 %) [93, 95, 101, 242–249, 255, 257] and nutria (5.8 %) [233] in France (fox pooled prevalence 13.9 %) (Table 7).

### Gaps and conclusions

Generally gaps were found in the literature regarding the following aspects (i) NUTS level specifications beyond the national level were absent in many reports, making it difficult to localise infection foci within specific areas for each country; (ii) many EU countries and ACs (*n* = 18) had no data on *E. multilocularis* prevalence in definitive or intermediate hosts, even in cases where *E. multilocularis* infection was probable because the parasite had been found in surrounding countries; (iii) data on the prevalence of the parasite in DHs, other than red foxes, and in some IHs were scarce and often reported in only one single study; (iv) the number of screened animals was considered insufficient in some reports in which the estimated prevalences were low; and (v) publication bias (for example there may be unpublished studies regarding the absence of *E. multilocularis* within the EU and/or ACs).

Furthermore, inadequacies were identified with regard to the assessment of *E. multilocularis* prevalence in red foxes. Specific gaps were that (i) the vast majority of studies were concentrated in six countries (Germany, France, Slovakia, Switzerland, Poland and Hungary, whereas the estimates of the pooled prevalence for other countries was based on few studies or further to this, in two cases (Liechtenstein and Spain) on single studies; (ii) sampling in some countries had been done in only specific areas in which it was assumed that the prevalence might be high and thus extrapolation of the data at national level could be biased; and (iii) bias may arise as a result of the sampling strategy used. The sampling strategy data for red foxes are summarised in Additional file 5: Table S1. In the current SR, 50/190 studies relating to fox sampling and *E. multilocularis* control programmes, excluding those based on coproELISA (*n* = 10), were included in the analysis. In addition, data were obtained from 20/190 papers describing rabies control programmes, in which foxes were probably mainly obtained by shooting. A further 38/190 papers included in this study did not report the type of sampling methods utilized. Additionally, in 133 studies examined, fox carcasses were made available to authors/authorities through other sources (road kill; hunting season). This type of sampling strategy can cause bias with regard to restrictions in sampling locations, since hunting for example is generally conducted in areas distant from human habitation. Therefore, in more than half of the prevalence studies, synanthropic fox populations living in villages, towns or cities were not included in the sampled animals. This may be the case for all fox sampling within the EU and ACs.

Specific gaps and weaknesses were also found for data relating to DHs other than red foxes. These were that (i) the number of studies was very low (*n* = 44) for the five DHs; (ii) some of the DHs are geographically restricted, for example, Arctic foxes are limited to northern latitudes [21, 24, 209] and golden jackals are found in only a few countries [24, 93, 209, 214]; (iii) some of the DHs such as raccoon dogs were not found on island countries (Ireland, Malta and the UK) and (iv) some of the DHs are protected species (e.g. the wolf). Specific gaps and deficiencies in data for IHs were that (i) the number of studies were very low (*n* = 27) for all screened IHs, excluding arvicolids and muskrats; and (ii) some of the IHs were geographically restricted.

In addition, in terms of the importance of definitive and intermediate hosts of *E. multilocularis*, this systematic review identified gaps regarding the following aspects: (i) the number of studies for the different hosts and the number of screened animals was very low, excluding red foxes, muskrats and arvicolids; and (ii) data on worm burden and worm maturity for the different DHs or fertility of protoscoleces in different IHs were lacking, precluding the assessment of the real role of each host in the maintenance of the life-cycle of *E. multilocularis*. However, the ranking of animals according to their importance as hosts may be useful in providing recommendations for the screening of DHs to better ascertain the presence of *E. multilocularis* in a given area. Host screening strategy should be as follows: in the absence of the most important DH, the second most important DH should be screened and so forth. Nevertheless, both the presence of hosts and the protected status of some species (e.g. wolves) are a matter to be taken into account when a recommendation for screening is given.

When conducting epidemiological studies, particularly if the absence of the parasite or a low to medium prevalence is expected and if red foxes cannot be screened, sylvatic animals should, preferably, be screened if the aim is to demonstrate the absence or presence of *E. multilocularis*. When the presence or maintenance of the life-cycle is to be assessed, the suitability of each DH to allow the full maturation of the parasite (worms producing infective eggs), and the evaluation of worm burden, should be taken into account. In a similar manner, when the presence or maintenance of the life-cycle needs to be assessed, the suitability of each IH to allow full maturation of the parasite (protoscolex production) should be considered.

The prevalence in muskrats and arvicolids seems to parallel those found in red foxes and if foxes cannot be screened, a larger number of muskrats and arvicolids than foxes would need to be screened to confirm the absence of *E. multilocularis*. This is necessary because the prevalence in foxes as compared to *Arvicola* spp. appear to correlate at a ratio of around 3:1. Similarly, in areas where both *M. glareolus* and *Microtus* spp, were found, *E. multilocularis* prevalence correlated with that in foxes at a ratio of 1:4–6 (Table 7). An exceptional case is Svalbard in Norway, where *Microtus* spp. had a 27 % *E. multilocularis* prevalence and the DH (Arctic fox) showed around 9 % prevalence [207, 209]. This could be attributed to ecological variables specific for this DH-IH interaction, since the IH (*Microtus levis*) has a very limited spatial distribution, while Arctic foxes are able to stroll on ice and can cover long distances and are therefore not limited to either the Spitsbergen Island nor to the Svalbard Archipelago. The only additional potential DH in this area is the dog, but this DH has to date not been screened in this region.

This SR has also highlighted gaps in our knowledge regarding mustelids and the role they may potentially play in the life-cycle of this parasite. Studies on *E. multilocularis* infection in mustelids (including *Mustela* spp., *Neovison vison*, *Lutra lutra*, *Meles meles* and *Martes* spp.) from Europe initially formed part of this meta-analysis. None of the studied mustelids from the Czech Republic (*n* = 6) [80], Denmark (*n* = 29) [24], Germany (*n* = 1142) [24, 122, 133, 142, 263], Poland (*n* = 22) [168, 169, 251], Slovakia (*n* = 18) [175, 177] and the Ukraine (*n* = 26) [77] were found infected with *E. multilocularis.* Interestingly, mustelids (*Martes* spp.) from Ryazan district, Russia were recently found to harbor adults of *E. multilocularis* [264]. As far as we are aware this is the only known report on the occurrence of *E. multilocularis* in mustelids and is the only known study that identified mustelids as ‘definitive hosts’ based on the presence of *E. multilocularis* adults in the intestine of 4/31 *Martes* species. While this infection can evidently occur, no information on *E. multilocularis* worm maturity, worm burden or prepatency was provided. Additionally, no mustelid-derived faecal samples have been unequivocally confirmed by molecular methods to be positive for *E. multilocularis.* Furthermore, we speculate that this infection may occur as a result of the predator-prey relationship of carnivorous mustelids and small rodents. In the absence of studies in which a larger number of mustelids are examined and/or experimental data we were reluctant to include data on mustelids in this analysis. Although the absence of *E. multilocularis* in mustelids in Europe suggests that they may not be important hosts of this parasite, further studies are required in order to clarify their role.

Importantly, the presence of *E. multilocularis* in red foxes cannot be excluded from countries where data may have been published using languages other than those represented by this SR consortium, but where this host is known to be present. For example there are many publications (albeit in Russian) on *E. multilocularis* in animals in the former Soviet Union ([265], Paul Torgerson personal communication]). High *E. mulitlocularis* infection rates in foxes (33.1 %) and raccoon dogs (15.4 %) were reported from Ryazan district [264] and in foxes from Bryansk Oblast (41 %) [266] and Kamchatka (14.7 %) in the east, respectively [267]. Similarly, high *E. multilocularis* infection rates of 40 % and 98 % were found in Arctic foxes from Krasnoyarsk region [268] and Sakha, Yakutia [269], respectively. In addition, reports on rodents have documented *E. multilocularis* infection in *Apodemus uralensis* and *Microtus arvalis* [270] in Kabardin-Balkar and *Clethrionomys* spp. in Sakha [271]. In a similar manner, the absence of *E. multilocularis* in foxes in countries for which only a few studies were available, may not be representative of the infection status of foxes in those particular areas.

## Conclusion

In conclusion, this SR confirmed the status of the red fox as the most important definitive host of *E. multilocularis* in the EU and ACs. If the prevalence in foxes was zero or low in a given area, there was no indication that the life-cycle of *E. multilocularis* was maintained by other DHs. In contrast, when the prevalence level in red foxes was greater than 3 %, both raccoon dogs and golden jackals, if present, seemed to play a similar role as the fox in the life-cycle of the parasite. In terms of IHs, muskrats and *M. glareolus*, if present, are important hosts in the life-cycle of *E. multilocularis*. Under specific conditions, *Arvicola* spp. and *Microtus* spp. could be important in the life-cycle of the parasite. Swine and insectivores seem to play no role in the life-cycle of *E. multilocularis* within the EU and ACs.

## Additional files

Additional file 1: Text S1. Questionnaire. (PDF 65 kb) Additional file 2: Text S2. Grey literature searching. (DOC 29 kb) Additional file 3: Text S3. List of the studies included in meta-analyses. (DOCX 47 kb) Additional file 4: Text S4. List of excluded studies. (DOCX 45 kb) Additional file 5: Table S1. Sampling strategy for red foxes (and Arctic foxes in Svalbard Islands, Norway). (DOC 66 kb)

## Abbreviations

AC: Adjacent countries
AE: Alveolar echinococcosis
AHAW: Animal health and welfare
BIOSIS: Biological Abstracts
CABI: Centre for Agricultural Bioscience International
CI: Confidence intervals
DH: Definitive host
EC: European Commission
EFSA: European Food Safety Authority
ELISA: Enzyme-linked immunosorbent assays
EMBASE: Excerpta Medica Database
EU: European Union
IH: Intermediate host
IST: Intestinal scraping technique
MEDLINE: MEDical Literature Analysis and Retrieval System
NOS: Newcastle-Ottawa scale
NUTS: Nomenclature of territorial units for statistics
PP: Pooled prevalence
SciSearch: Science Citation Index
SCT: Sedimentation and counting technique
SR: Systematic review
STN: Scientific and Technical Information Network International
