# Supplementary material for: The geographical distribution and prevalence of Echinococcus multilocularis in animals in the European Union and adjacent countries: a systematic review and meta-analysis
Source: Parasit Vectors. 2016 Sep 28;9:519. doi: 10.1186/s13071-016-1746-4 (PMC5039905; doi:10.1186/s13071-016-1746-4)
Supplement: Additional file 1: Text S1. — Questionnaire. (PDF 65 kb) [file 13071_2016_1746_MOESM1_ESM.pdf]

QUESTIONNAIRE on:

"*Echinococcus multilocularis* infection in animals"

Introduction to the questionnaire

Dear Colleague,

the European Food Safety Authority (EFSA) is funding a EU project on "***Echinococcus multilocularis* infection in animals**" in order to revise the **EU regulation No 1152/2011** in light of scientific developments.
In order to be able to provide a comprehensive and quantitative assessment of EM infections in animals, it is important to identify and collect data on all relevant aspects on the epidemiology and risk factors for the prevention and control of this disease. This inventory should also include data available from **Member States** that would be of relevance for **systematic reviews** approach of literature and data.
In order to support EFSA in this project we would be glad to ask you to provide the information required in the following questionnaire.

You can send back this **Questionnaire** by email, fax or ordinary mail at the following address:

Dr. Adriano Casulli
EURLP
MIPI department
Istituto Superiore di Sanità
Viale Regian Elena 299
00161 Rome, Italy

Email: adriano.casulli@iss.it
Fax: +39 06 4990 3561

For any request please do not hesitate to contact: adriano.casulli@iss.it

A1: DATA ON COMPILER

1. First name and Surname:

2. Position:

2. Affiliation (Institution and address):

3. Contacts (e-mail, telephone, fax):

A2: OTHER SUBJECTS (institutions or persons)

If you are aware of other persons that could contribute to the provision of the information requested, please fill in this section in order to give us the opportunity to contact them (replicate this information for any of the subject that you include here).

1. First name and Surname:

2. Position:

3. Affiliation (Institution and address):

4. Contacts (e-mail, telephone, fax):

B1: INFORMATION SOURCES

Are information on *Echinococcus multilocularis/* Alveolar echinococcosis in any of these sources available? (specify if in English or in national language)

B1.1 Proceedings of National meetings: No ☐ Yes ☐ (specify title/s)

____

B1.2 Reports from University and National Institutes (Veterinary or Medical): No ☐ Yes ☐ (specify title/s)

____

B1.3 Master and PhD thesis: No ☐ Yes ☐ (specify title/s)

____

B1.4 Reports on National/Regional projects: No ☐ Yes ☐ (specify title/s)

____

B1.5 Articles published in National Journals: No ☐ Yes ☐ (specify title/s)

____

B1.6 Reports on surveillance, monitoring, diagnostic and control activities on wild and domestic animals and humans: No ☐ Yes ☐ (if Yes, please specify in box **B2**)

B2: ADDITIONAL INFORMATION SOURCES
Surveillance, monitoring, diagnostic and control activities on *Echinococcus multilocularis*/ Alveolar echinococcosis.

2.1 Monitoring: No ☐ Yes ☐ (if yes please specify)

____

Data available from (year): __________ to __________
Species involved (humans, foxes, dogs, rodents, others): ______________________________
Institutions involved:____

2.2 Surveillance: No ☐ Yes ☐ (if yes please specify)

____

Data available from (year): __________ to __________
Species involved (humans, foxes, dogs, rodents, others): ______________________________
Institutions involved:____

2.3 Diagnosis: No ☐ Yes ☐ (if yes please specify)

____

Data available from (year): __________ to __________
Species involved (humans, foxes, dogs, rodents, others): ______________________________
Institutions involved:____

2.4 Control: No ☐ Yes ☐ (if yes please specify)

____

Data available from (year): __________ to __________
Species involved (humans, foxes, dogs, rodents, others): ______________________________
Institutions involved:____

2.5 Other: No ☐ Yes ☐ (if yes please specify)

____

Data available from (year): __________ to __________
Species involved (humans, foxes, dogs, rodents, others): ______________________________
Institutions involved:____

B3: DATA SHARING WITH EFSA

Is there the possibility to share data or provide the access to databases on *Echinococcus multilocularis/* Alveolar echinococcosis in any of these forms?

B3.1 Aggregated form (summary data, tables, reports): No ☐ Yes ☐ (specify)

____

B3.2 Punctual data (single records, databases): No ☐ Yes ☐ (specify)

____

COMMENTS OR ADDITIONAL INFORMATION

Please provide any other comment or information about the availability of data on *Echinococcus multilocularis/* Alveolar echinococcosis in your country)
