# Supplementary material for: The geographical distribution and prevalence of Echinococcus multilocularis in animals in the European Union and adjacent countries: a systematic review and meta-analysis
Source: Parasit Vectors. 2016 Sep 28;9:519. doi: 10.1186/s13071-016-1746-4 (PMC5039905; doi:10.1186/s13071-016-1746-4)
Supplement: Additional file 2: Text S2. — Grey literature searching. (DOC 29 kb) [file 13071_2016_1746_MOESM2_ESM.doc]

S2 Text.

Grey literature searching

Bachelor, Masters and PhD theses searches were carried out using the keywords “*Echinococcus multilocularis*” and “alveolar echinococcosis” and the following databases (available online):

- http://ethos.bl.uk/Home.do
- http://www.dart-europe.eu/basic-search.php
- https://www.daad.de/deutschland/promotion/phd/en/13306-phdgermany-database/
- https://catalogue.lse.ac.uk/Record/1149203
- http://www.theses.fr/
- http://biblioteca.ucm.es/
- http://digital.csic.es/
- https//www.tesisenred.net/
- http://www.proquest.com/en-US/catalogues/databases/detail/abi_inform.shtml
- https://portal.dnb.de/opac.htm
- http://www.sudoc.abes.fr/
- http://www.collectionscanada.gc.ca/thesescanada/
- http://library.stanford.edu/guides/find-dissertations-and-theses
- http://www.oclc.org/support/services/firstsearch/documentation/dbdetails/details/WorldCatDis sertations.en.html
- <https://qspace.library.queensu.ca/handle/1974/290>.
