# Supplementary material for: The geographical distribution and prevalence of Echinococcus multilocularis in animals in the European Union and adjacent countries: a systematic review and meta-analysis
Source: Parasit Vectors. 2016 Sep 28;9:519. doi: 10.1186/s13071-016-1746-4 (PMC5039905; doi:10.1186/s13071-016-1746-4)
Supplement: Additional file 3: Text S3. — List of the studies included in meta-analyses. (DOCX 47 kb) [file 13071_2016_1746_MOESM3_ESM.docx]

**S3 Text: List of the studies included in meta-analyses**

Ahlman VP. Epidemiological studies on the occurrence of rabies and little tapeworm *Echinococcus multilocularis* in Saarland. PhD Thesis Freien University Berlin. 1997.

Al-Sabi MNS, Chriel M, Enemark HL. Endoparasites of the raccoon dog (*Nyctereutes procyonoides*) and the red fox (*Vulpes vulpes*) in Denmark 2009-2012 - A comparative study. International journal for parasitology Parasites and wildlife. 2013;2:144-51.

Anonymous. Alveolar echinococcosis. Prevalence of *Echinococcus multilocularis* in foxes. Releve epidemiologique hebdomadaire / Section d'hygiene du Secretariat de la Societe des Nations = Weekly epidemiological record / Health Section of the Secretariat of the League of Nations. 1993;68:165-8.

Antolova D, Miterpakova M, Reiterova K, Dubinsky P. Influence of anthelmintic baits on the occurrence of causative agents of helminthozoonoses in red foxes (*Vulpes vulpes*) . Helminthologia. 2006;43:226-31.

Antolova D, Reiterova K, Miterpakova M, Dinkel A, Dubinsky P. The first finding of *Echinococcus multilocularis* in dogs in Slovakia: an emerging risk for spreading of infection. Zoonoses and Public Health. 2009;56(2):53-8.

Antolova D, Miterpakova M, Radonak J, Hudackova D, Szilagyiova M, Zacek M. Alveolar echinococcosis in a highly endemic area of Northern Slovakia between 2000 and 2013. Euro surveillance : bulletin Europeen sur les maladies transmissibles = European communicable disease bulletin. 2014;19(34).

Aubert M, Jacquier P, Artois M, Barrat MJ, Basile AM. Parasitism of red fox (*Vulpes vulpes*) by *Echinococcus multilocularis* in Lorraine (France) and their consequences on human contamination. Recueil De Medecine Veterinaire. 1987;163(10):839-43.

Aubert M, Jacquier P, Artois M. Hosts of *Echinococcus multilocularis* in Lorraine and their consequences on human contamination. I. Biogeographic approach. Bulletin de la Societe Francaise de Parasitologie. 1986;4:59-64.

Bagrade G, Snabel V, Romig T, Ozolins J, Huettner M, Miterpakova M, et al. *Echinococcus multilocularis* is a frequent parasite of red foxes (*Vulpes vulpes*) in Latvia. Helminthologia. 2008;45(4):157-61.

Bagrade G, Kirjusina M, Vismanis K, Ozolins J. Helminth parasites of the wolf *Canis lupus* from Latvia. Journal of helminthology 2009;83:63-8.

Ballek D. Occurrence of *Echinococcus multilocularis* and other cestodes and nematodes in the red fox (*Vulpes vulpes*) in the administrative districts Arnsberg, Detmold and Kassel. PhD Thesis. Hannover: Tieraerztliche Hochschule Hannover. 1991.

Ballek D, Takla M, Ising-Volmer S, Stoye M. The helminth fauna of red foxes (*Vulpes vulpes* Linnaeus 1758) in north Hesse and east Westphalia. 1. Cestodes. Deutsche Tierarztliche Wochenschrift. 1992;99:362-5.

Baudouin MC, Aubert MFA. *Echinococcus multilocularis* Leuckart, 1863 in foxes (*Vulpes vulpes* Linnaeus, 1758) in the Vosges: a parasite dangerous to man. Revue Scientifique et Technique - Office International des Epizooties. 1993;12:161-3.

Baumeister S, Pohlmeyer K, Kuschfeldt S, Stoye M. On the prevalence of *Echinococcus multilocularis* and other metacestodes and cestodes in the muskrat (*Ondatra zibethicus* Link, 1795) in Lower Saxony. Deutsche Tierarztliche Wochenschrift. 1997;104(10):448-52.

Berke O, von Keyserlingk M. Increase of the prevalence of *Echinococcus multilocularis* infection in red foxes in Lower Saxony. Deutsche Tierarztliche Wochenschrift. 2001;108(5):201-5.

Berke O, von Keyserlingk M, Broll S, Kreienbrock L. On the distribution of *Echinococcus multilocularis* in red foxes in Lower Saxony: identification of a high risk area by spatial epidemiological cluster analysis. Berliner Und Munchener Tierarztliche Wochenschrift. 2002;115(11-12):428-34.

Berke O, Romig T, von Keyserlingk M. Emergence of *Echinococcus multilocularis* among red foxes in northern Germany, 1991-2005. Veterinary Parasitology. 2008;155(3-4):319-22.

Bilger B, Veit P, Muller V, Merckelbach A, Kersten D, Stoppler H, et al. Further-studies of *Echinococcus multilocularis* infection of the red fox in the district of Tubingen. Tierarztliche Umschau. 1995;50(7):465-70.

Bonnin JL, Artois M, Aubert M. Incidence and distribution of larval cestode infections in rodents in Lorraine. Revue de Medecine Veterinaire. 1989;140:589-97.

Borecka A, Gawor J, Malczewska M, Malczewski A. Prevalence of *Echinococcus multilocularis* tapeworm in red foxes in central Poland. Medycyna Wet. 2007;63(11):1333-5.

Borecka A, Gawor J, Malczewska M, Malczewski A. Occurence of *Echinococcus multilocularis* in red foxes (*Vulpes vulpes*) in southern Poland. Helminthologia. 2008;45(1):24-7.

Borecka A, Gawor J, Malczewska M, Malczewski A. Prevalence of zoonotic helminth parasites of the small intestine in red foxes from central Poland. Medycyna Weterynaryjna. 2009;65:33-5.

Borgsteede FHM, Tibben JH, van der Giessen JWB. The muskrat (*Ondatra zibethicus*) as intermediate host of cestodes in the Netherlands. Veterinary parasitology 2003; 117:29-36.

Boussinesq M, Bresson S, Liance M, Houin R. A new natural intermediate host of *E. multilocularis* in France - the muskrat (*Ondatra zibethicus*). Annales De Parasitologie Humaine Et Comparee. 1986;61(4):431-4.

Brochier B, Coppens P, Losson B, Aubert MFA, Bauduin B, Barrat MJ, et al. Prevalence of *Echinococcus multilocularis* infestation in the red fox (*Vulpes Vulpes*) in the province of Luxembourg (Belgium) - a preliminary survey. Annales De Medecine Veterinaire. 1992;136(7):497-501.

Brochier B, De Blander H, Hanosset R, Berkvens D, Losson B, Saegerman C. *Echinococcus multilocularis* and *Toxocara canis* in urban red foxes (*Vulpes vulpes*) in Brussels, Belgium. Preventive Veterinary Medicine. 2007;80(1):65-73.

Brossard M, Andreutti C, Siegenthaler M. Infection of red foxes with *Echinococcus multilocularis* in western Switzerland. Journal of helminthology. 2007;81(4):369–76.

Bruzinskaite R, Marcinkute A, Strupas K, Sokolovas V, Deplazes P, Mathis A, et al. Alveolar echinococcosis , Lithuania. Emerging Infectious Diseases. 2007;13:1618-9.

Bruzinskaite R, Sarkunas M, Torgerson PR, Mathis A, Deplazes P. Echinococcosis in pigs and intestinal infection with *Echinococcus* spp. in dogs in southwestern Lithuania. Veterinary Parasitology. 2009;160(3-4):237-41.

Bruzinskaite-Schmidhalter R, Sarkunas M, Malakauskas A, Mathis A, Torgerson PR, Deplazes P. Helminths of red foxes (*Vulpes vulpes*) and raccoon dogs (*Nyctereutes procyonoides*) in Lithuania. Parasitology. 2012;139:120-7.

Burlet P, Deplazes P, Hegglin D. Age, season and spatio-temporal factors affecting the prevalence of *Echinococcus multilocularis* and *Taenia taeniaeformis* in *Arvicola terrestris*. Parasites & Vectors. 2011;4.

Cada F, Martinek K, Kolarova L. Cats (*Felis catus f. dom*.) as definitive host of *Echinococcus multilocularis*. Veterinarstvi. 1999;49(1):6–7.

Calderini P, Magi M, Gabrielli S, Brozzi A, Kumlien S, Grifoni G, et al. Investigation on the occurrence of *Echinococcus multilocularis* in Central Italy. Bmc Veterinary Research. 2009;5.

Casulli A, La Rosa G, Manfredi MT, Di Cerbo AR, Dinkel A, Romig T, et al. Copro-diagnosis of *Echinococcus multilocularis* by a nested PCR in red foxes (*Vulpes vulpes*) from northern Italy. Parassitologia. 2004;46(4):419-20.

Casulli A, Manfredi MT, La Rosa G, Di Cerbo AR, Dinkel A, Romig T, et al. *Echinococcus multilocularis* in red foxes (*Vulpes vulpes*) of the Italian Alpine region: is there a focus of autochthonous transmission? International Journal for Parasitology. 2005;35(10):1079-83.

Casulli A, Szell Z, Pozio E, Sreter T. Spatial distribution and genetic diversity of *Echinococcus multilocularis* in Hungary. Veterinary Parasitology. 2010;174(3-4):241-6.

Combes B, Comte S, Raton V, Raoul F, Boue F, Umhang G, et al. Westward spread of *Echinococcus multilocularis* in foxes, France, 2005-2010. Emerging Infectious Diseases. 2012;18(12):2059-62.

Coudert J, Euzeby J, Garin JP. Incidence of *E. multilocularis* in common fox (*Vulpes vulpes*) in the nord-east of France. Lyon medical. 1970;32:293-8.

Davidson RK, Oines O, Madslien K, Mathis A. *Echinococcus multilocularis* adaptation of a worm egg isolation procedure coupled with a multiplex PCR assay to carry out large-scale screening of red foxes (*Vulpes vulpes*) in Norway. Parasitology Research. 2009;104(3):509-14.

Davidson R, Oines O, Albin-Amiot C, Hopp P, Madslien K, Hagstrom A, et al. Ghost- hunting is *Echinococcus multilocularis* really absent from mainland Norway? Tropical Medicine & International Health. 2013;18:97.

Deblock S, Petavy AF. Hepatic larvae of Cestode parasites of the vole rat *Arvicola terrestris* in Auvergne (France). Annales de parasitologie humaine et comparee. 1983;58:423-37.

Deblock S, Petavy AF, Gilot B. Intestinal helminths of the red fox *Vulpes vulpes* L. in the Massif Central France. Canadian Journal of Zoology. 1988;66:1562-9.

Delattre P, Pascal M, Damange JP. Towards a strategy for the epidemiological study of alveolar echinococcosis. Apropos of cases of infestation seen in *Microtus arvalis* P. in the Doubs (France). Annales de parasitologie humaine et comparee. 1985;60(4):389–405.

Delattre P, Pascal M, Lepesteur MH, Giraudoux P, Damange JP. Ecological and epidemiological characteristics of *Echinococcus multilocularis* during a complete population-cycle in a secondary host (*Microtus arvalis*). Canadian Journal of Zoology-Revue Canadienne De Zoologie. 1988;66(12):2740-50.

Delattre P, Giraudoux P, Quere JP. Epidemiologic consequences of the receptivity of a new intermediate host of *Echinococcus multilocularis*, and of the space-time localization of the infected rodents. Comptes Rendus De L Academie Des Sciences Serie Iii-Sciences De La Vie-Life Sciences. 1990;310(8):339-44.

Denzin N, Schliephake A, Ewert B. *Echinococcus multilocularis* in red foxes in Saxony-Anhalt: Identification of areas of increased risk of infestation and association of the infestation probability with the average annual maximum temperature. Berliner Und Munchener Tierarztliche Wochenschrift. 2005;118(9-10):404-9.

Denzin N, Schliephake A, Wirth A. Spatiotemporal analysis of the infection of the red fox (*Vulpes vulpes* L.) with *Echinococcus multilocularis* in Saxony-Anhalt. Berliner Und Munchener Tierarztliche Wochenschrift. 2009;122(3-4):82-92.

Denzin N, Schliephake A, Froehlich A, Ziller M, Conraths FJ. On the Move? *Echinococcus multilocularis* in red foxes of Saxony-Anhalt (Germany). Transboundary and Emerging Diseases. 2014;61(3):239-46.

Deplazes P, Gloor S, Stieger C, Hegglin D. Urban transmission of *Echinococcus multilocularis*. In: Craig P, Pawlowski Z, editors. Cestode Zoonoses: Echinococcosis and Cysticercosis: An Emergent and Global Problem. Nato Science Series, Sub-Series I: Life and Behavioural Sciences. 2002;341:287-97.

Deplazes P, Hegglin D, Gloor S, Romig T. Wilderness in the city: the urbanization of *Echinococcus multilocularis*. Trends in Parasitology. 2004;20(2):77-84.

Deutz A, Fuchs K, Lassnig H, Hinterdorfer F. Prevalence of *E. multilocularis* in foxes in Styria taking into consideration biometrical methods. Berliner und Munchener tierarztliche Wochenschrift. 1995;108:408-11.

Di Cerbo AR, Manfredi MT, Trevisiol K, Bregoli M, Ferrari N, Pirinesi F, et al. Intestinal helminth communities of the red fox (*Vulpes vulpes* L.) in the Italian Alps. Acta Parasitologica. 2008;53:302-11.

Dinkel A, von Nickisch-Rosenegk M, Bilger B, Merli M, Lucius R, Romig T. Detection of *Echinococcus multilocularis* in the definitive host: Coprodiagnosis by PCR as an alternative to necropsy. Journal of Clinical Microbiology. 1998;36(7):1871-6.

Dubinsky P, Svobodova V, Turcekova L, Literak I, Martinek K, Reiterova K, et al. *Echinococcus multilocularis* in Slovak Republic: The first record in red foxes (*Vulpes vulpes*). Helminthologia. 1999;36(2):105-10.

Dubinsky P, Varady M, Reiterova K, Miterpakova M, Turcekova L. Prevalence of *Echinococcus multilocularis* in red foxes in the Slovak Republic. Helminthologia. 2001;38(4):215-9.

Dubinsky P, Malczewski A, Miterpakova M, Gawor J, Reiterova K. *Echinococcus multilocularis* in the red fox *Vulpes vulpes* from the East Carpathian region of Poland and the Slovak Republic. Journal of Helminthology. 2006;80(3):243-7.

Duscher G, Steineck T, Gunter P, Prosl H, Joachim A. *Echinococcus multilocularis* in foxes in Wien and surrounding territories. Wiener Tierarztliche Monatsschrift. 2005;92(1):16-20.

Duscher G, Prosl H, Joachim A. Scraping or shaking-a comparison of methods for the quantitative determination of *Echinococcus multilocularis* in fox intestines. Parasitology Research. 2005;95(1):40–2.

Dyachenko V, Pantchev N, Gawlowska S, Vrhovec MG, Bauer C. *Echinococcus multilocularis* infections in domestic dogs and cats from Germany and other European countries. Veterinary Parasitology. 2008;157(3-4):244-53.

Eckert J, Deplazes P, Ewald D, Gottstein B. Parasitological and immunological methods for the detection of *Echinococcus multilocularis* in foxes Mitteilungen der Oesterreichischen Gesellschaft fuer Tropenmedizin und Parasitologie. 1991;13:25-30.

EFSA. The Community summary Report on Trends and Sources of Zoonoses, Zoonotic Agents, antimicrobial resistance andand Food-borne Ourbreaks in the European Union in 2005. EFSA Journal. 2006;94:2-228.

EFSA. The Community summary Report on Trends and Sources of Zoonoses, Zoonotic Agents, antimicrobial resistance andand Food-borne Ourbreaks in the European Union in 2006. EFSA Journal. 2007;130:2-352.

EFSA. The EU Summary Report on Trends and Sources of Zoonoses, Zoonotic Agents and Fodd-borne Outbreaks in 2011. EFSA Journal. 2013;11:3129.

EFSA. Assessment of *Echinococcus multilocularis* surveillance reports submitted 2013 in the context of Commission Regulation (EU) No 1152/2011. EFSA Journal. 2013;11:3465pp.

EFSA. Assessment of *Echinococcus multilocularis* surveillance reports submitted in 2014 in the context of Commission Regulation (EU) No 1152/2011. EFSA Journal. 2014;12:3875.

EFSA. The EU summary Report on Trends and Sources of Zoonoses, Zoonotic Agents and Food-borne Outbreaks in 2013. EFSA Journal. 2015;13:3991.

EFSA. Assessment of Echinococcus multilocularis surveillance data 2012-2013 submitted by Norway in the context of Commission Regulation (EU) No 1152/2011. EFSA Journal. 2015;13:4035.

Enemark HL, Al-Sabi MN, Knapp J, Staahl M, Chriel M. Detection of a high-endemic focus of *Echinococcus multilocularis* in red foxes in southern Denmark, January 2013. Euro surveillance : bulletin Europeen sur les maladies transmissibles = European communicable disease bulletin. 2013;18(10):20420pp.

Eskens U. On the occurrence of *Echinococcus multilocularis* in red fox in the areas adjoining the State Medical, Food and Veterinary Investigation Bure in Mid-Hessen. Zeitschrift fuer Jagdwissenschaft. 1997;43:154-65.

Ewald D. Distribution of the tapeworm *Echinococcus multilocularis* in the fox *Vulpes vulpes* and muskrat *Ondatra zibethicus* in the Freiburg administrative district. Mitteilungen des Badischen Landesvereins fuer Naturkunde und Naturschutz. 1990;15:81-100.

Ewald D, Eckert J, Gottstein B, Straub M, Nigg H. Parasitological and serological studies on the prevalence of *Echinococcus multilocularis* Leuckart, 1863 in red foxes (*Vulpes vulpes* Linnaeus, 1758) in Switzerland. Revue scientifique et technique (International Office of Epizootics). 1992;11(4):1057-61.

Ewald D, Eckert J. Distribution and frequency of *Echinococcus multilocularis* among red fox in north, South and East Switzerland as well as in the principality of Liechtenstein. Zeitschrift Fur Jagdwissenschaft. 1993;39(3):171-80.

Fesseler M, Schott E, Mueller B. Occurrence of *Echinococcus multilocularis* among cats in the Tuebingen region of the Federal Republic of Germany. Tieraerztliche Umsch. 1989;44: 766-75.

Frank B, Zeyhle E. *Echinococcus* and other tapeworm larvae in muskrat (*Ondatra zibethicus*). Nachrichtenblatt des Deutschen Pflanzenschutzdienstes. 1981 ;33:166–170.

Franssen F, Nijsse R, Mulder J, Cremers H, Dam C, Takumi K, et al. Increase in number of helminth species from Dutch red foxes over a 35-year period. Parasites & Vectors. 2014;7.

Friedland T, Steiner B, Boeckeler W. Prevalence of cysticercosis in muskrats Ondatra zibethica in schleswig-holstein west Germany. . Zeitschrift fuer Jagdwissenschaft. 1985;31:134-9.

Gottstein B, Deplazes P, Eckert J, Muller B, Schott E, Helle O, et al. Serological (Em2-ELISA) and parasitological examinations of fox populations for *Echinococcus multilocularis* infections. Zentralblatt fur Veterinarmedizin Reihe B Journal of veterinary medicine Series B. 1991;38(3):161-8.

Gottstein B, Saucy F, Wyss C, Siegenthaler M, Jacquier P, Schmitt M, et al. Investigations on a Swiss area highly endemic for *Echinococcus multilocularis*. Applied parasitology. 1996;37(2):129-36.

Gottstein B, Saucy F, Deplazes P, Reichen J, Demierre G, Busato A, et al. Is high prevalence of *Echinococcus multilocularis* in wild and domestic animals associated with disease incidence in humans? Emerging Infectious Diseases. 2001;7(3):408-12.

Goutal-Rotszyld C. Contribution to the study of internal parasitism of foxes (*Vulpes vulpes*) in middle Pyrenees: search for *Echinococcus multilocularis*. Theses University of Toulouse. 2005.

Guerra D, Hegglin D, Bacciarini L, Schnyder M, Deplazes P. Stability of the southern European border of *Echinococcus multilocularis* in the Alps: evidence that *Microtus arvalis* is a limiting factor. Parasitology. 2014;141(12):1593-602.

Guislain M-H, Raoul F, Giraudoux P, Terrier M-E, Froment G, Ferte H, et al. Ecological and biological factors involved in the transmission of *Echinococcus multilocularis* in the French Ardennes. Journal of Helminthology. 2008;82(2):143-51.

Hanosset R, Saegerman C, Adant S, Massart L, Losson B. *Echinococcus multilocularis* in Belgium: Prevalence in red foxes (*Vulpes vulpes*) and in different species of potential intermediate hosts. Veterinary Parasitology. 2008;151(2-4):212-7.

Hartel KS, Spittler H, Doering H, Winkelmann J, Hoerauf A, Reiter-Owona I. The function of wild nutria (*Myocastor coypus*) as intermediate hosts for *Echinococcus multilocularis* in comparison to wild muskrats (*Ondatra zibethicus*). International Journal of Medical Microbiology. 2004;293:62-3.

Hegglin D, Ward PI, Deplazes P. Anthelmintic baiting of foxes against urban contamination with *Echinococcus multilocularis*. Emerging Infectious Diseases. 2003;9(10):1266-72.

Henttonen H, Fuglei E, Gower CN, Haukisalmi V, Ims RA, Niemimaa J, et al. *Echinococcus multilocularis* on Svalbard: introduction of an intermediate host has enabled the local life cycle. Parasitology. 2001;123:547-52.

Hofer S, Gloor S, Bontadina F, Mathis A, Hegglin D, Mueller U, et al. Life cycle of *Echinococcus multilocularis* in the City of Zurich: A new risk? Schweizerische Medizinische Wochenschrift. 1999;129:1125.

Hofer S, Gloor S, Muller U, Mathis A, Hegglin D, Deplazes P. High prevalence of *Echinococcus multilocularis* in urban red foxes (*Vulpes vulpes*) and voles (*Arvicola terrestris*) in the city of Zurich, Switzerland. Parasitology. 2000;120:135-42.

Houin R, Deniau M, Liance M. *Arvicola terrestris* (L) 1758 1st rodent found naturally infested with *Echinococcus multilocularis* Leuckart, 1863, in France. Comptes Rendus Hebdomadaires Des Seances De L Academie Des Sciences Serie D. 1980;290(19):1269-71.

Houin R, Deniau M, Liance M, Puel F. Arvicola terrestris an intermediate host of *Echinococcus multilocularis* in France - epidemiological consequences. International Journal for Parasitology. 1982;12(6):593-600.

Hurnikova Z, Miterpakova M, Chovancova B. The important zoonoses in the protected areas of the Tatra National Park (TANAP). Wiadomosci parazytologiczne. 2009;55:395-8.

Immelt U, Thelen U, Eskens U. Investigation of *Echinococcus multilocularis* in red foxes and their possible relationship to human alveolar echinococcosis. Tieraerztliche Umschau. 2009;64(4):199-212.

Isaksson M, Hagstrom A, Armua-Fernandez MT, Wahlstrom H, Agren EO, Miller A, et al. A semi-automated magnetic capture probe based DNA extraction and real-time PCR method applied in the Swedish surveillance of *Echinococcus multilocularis* in red fox (*Vulpes vulpes*) faecal samples. Parasites & Vectors. 2014;7:583.

Janka S, Stoye M. Studies on *Echinococcus multilocularis* and *Trichinella spiralis* infections in the red fox in the Karlsruhe area. Tierarztliche Umschau. 1998;53(4):221-6.

Janko C, Linke S, Schroeder W, Koenig A, Romig T, Thoma D. Infection pressure of human alveolar echinococcosis due to village and small town foxes (*Vuples vulpes*) living in close proximity to residents. European Journal Of Wildlife Research. 2011;57:1033-42.

Jonas D, Hahn W. Evidence of *Echinococcus multilocularis* in foxes in Rheinland-Pfalz. Praktische Tierarzt. 1984;65:65,7-9.

Jonas D, Drager K. Investigation of *Echinococcus multilocularis* infection in foxes: development since 1982 and the situation in 1996/97 in Rhineland Palatinate. Tierarztliche Umschau. 1998;53:214,7-21.

Karamon J, Ziomko I, Cencek T, Sroka J, Zieba P. Prevalence of *Echinococcus multilocularis* in red foxes in the Lublin voivodeship, Poland: preliminary study. Medycyna Weterynaryjna. 2008;64: 1237-9.

Karamon J, Sroka J, Cencek T, Michalski MM, Zieba P, Karwacki J. Prevalence of *Echinococcus multilocularis* in red foxes in two eastern provinces of Poland. Bulletin of the Veterinary Institute in Pulawy. 2011;55(3):429-33.

Karamon J, Kochanowski M, Sroka J, Cencek T, Rozycki M, Chmurzynska E, et al. The prevalence of *Echinococcus multilocularis* in red foxes in Poland-current results (2009-2013). Parasitology Research. 2014;113(1):317-22.

Kharchenko VA, Kornyushin VV, Varodi EI, Malega OM. Occurrence of *Echinococcus multilocularis* (Cestoda, Taeniidae) in red foxes (*Vulpes vulpes*) from Western Ukraine. Acta Parasitologica. 2008;53(1):36-40.

Kikkert PF. Detection of *Echinococcus multilocularis* in red fox (*Vulpes vulpes*) in The Netherlands at the border with Germany. Thesis. 2011.

Knapp J, Guislain MH, Bart JM, Raoul F, Gottstein B, Giraudoux P, et al. Genetic diversity of *Echinococcus multilocularis* on a local scale. Infection Genetics and Evolution. 2008;8(3):367-73.

Koenig A, Romig T, Thoma D, Kellermann K. Drastic increase in the prevalence in *Echinococcus multilocularis* in foxes (*Vulpes vulpes*) in southern Bavaria, Germany. European Journal of Wildlife Research. 2005;51(4):277-82.

Koenig A, Romig T, Janko C, Hildenbrand R, Holzhofer E, Kotulski Y, Ludt C, Merli M, Eggenhofer S, Thoma D, Vilsmeier J, Zannantonio D. Integrated-baiting concept against *Echinococcus multilocularis* in foxes is successful in southern Bavaria, Germany. European Journal of Wildlife Research. 2008;54(3):439–47.

Koenig A, Romig T. Fox tapeworm *Echinococcus multilocularis*, an underestimated threat: a model for estimating risk of contact. Wildlife Biology. 2010;16(3):258-66.

Kolarova L, Pavlasek I, Chalupsky J. *Echinococcus multilocularis* Leuckart, 1863 in the Czech Republic. Helminthologia. 1996;33(2):59-65.

Kornyushin VV, Malyshko EI, Malega AM. The helminths of wild predatory mammals of Ukraine. Cestodes. Vestnik Zoologii. 2011;45:483-90.

Lassnig H, Prosl H, Hinterdorfer F. Parasites of the red fox (*Vulpes vulpes*) in Styria. Wiener Tierarztliche Monatsschrift. 1998;85:116-22.

Laurimaa L, Davison J, Plumer L, Süld K, Oja R, Moks E, Keis M, Hindrikson M, Kinkar L, Laurimäe T, Abner J, Remm J, Anijalg P, Saarma U. Noninvasive detection of *Echinococcus multilocularis* tapeworm in urban area, Estonia. Emerging Infectious Disease. 2015;21:163–4.

Laurimaa L, Sueld K, Moks E, Valdmann H, Umhang G, Knapp J, et al. First report of the zoonotic tapeworm *Echinococcus multilocularis* in raccoon dogs in Estonia, and comparisons with other countries in Europe. Veterinary Parasitology. 2015;212(3-4):200-5.

Learmount J, Zimmer IA, Conyers C, Boughtflower VD, Morgan CP, Smith GC. A diagnostic study of *Echinococcus multilocularis* in red foxes (*Vulpes vulpes*) from Great Britain. Veterinary Parasitology. 2012;190(3-4):447-53.

Letkova V, Lazar P, Curlik J, Goldova M, Kocisova A, Kosuthova L, et al. The red fox (*Vulpes vulpes* L.) as a source of zoonoses. Veterinarski Arhiv. 2006;76:73-81.

Letkova V, Lazar P, Soroka J, Goldova M, Curlik J. Epizootiology of game cervid cysticercosis. Natura Croatica. 2008;17:311-8.

Loos-Frank B. Larval cestodes In Southwest German rodents. Zeitschrift fuer Angewandte Zoologie. 1987;74:97-106.

Losson B, Mignon B, Brochier B, Bauduin B, Pastoret PP. *Echinococcus multilocularis* infection in the red fox (*Vulpes vulpes*) in the province of Luxembourg (Belgium): Results of a survey conducted between 1993-1995. Annales De Medecine Veterinaire. 1997;141(2):149-53.

Losson B, Kervyn T, Detry J, Pastoret PP, Mignon B, Brochier B. Prevalence of *Echinococcus multilocularis* in the red fox (*Vulpes vulpes*) in southern Belgium. Veterinary Parasitology. 2003;117(1-2):23-8.

Lucius R, Boeckeller W, Pfeiffer AS. Parasitic infestation of the domestic and wild animals of Schleswig-Holstein West Germany parasites of the inner organs of red fox (Vulpes vulpes). Zeitschrift fuer Jagdwissenschaft. 1988;34:242-55.

Maas M, Dam-Deisz WDC, van Roon AM, Takumi K, van der Giessen JWB. Significant increase of *Echinococcus multilocularis* prevalence in foxes, but no increased predicted risk for humans. Veterinary Parasitology. 2014;206(3-4):167-72.

Machnicka-Rowinska B, Rocki B, Dziemian E, Kolodziej-Sobocinska M. Raccoon dog (*Nyctereutes procyonoides*) the new host of *Echinococcus multilocularis* in Poland. Wiadomosci parazytologiczne. 2002;48(1):65-8.

Machnicka B, Dziemian E, Rocki B, Kolodziej-Sobocinska M. Detection of *Echinococcus multilocularis* antigens in faeces by ELISA. Parasitology Research. 2003;91(6):491-6.

Madslien K, Davidson R, Handeland K, Oines O, Urdahl AM, Hopp P. The surveillance and control programme for *Echinococcus multilocularis* in red foxes (*Vulpes vulpes*) in Norway. Annual Report 2011. 2011.

Magi M, Macchioni F, Dell'Omodarme M, Prati MC, Dell'Omodarme M, Calderini P, et al. Endoparasites of red fox (*Vulpes vulpes*) in Central Italy. Journal Of Wildlife Diseases. 2009;45:881-5.

Magnaval J-F, Boucher C, Morassin B, Raoul F, Duranton C, Jacquiet P, et al. Epidemiology of alveolar echinococcosis in southern Cantal, Auvergne region, France. Journal of Helminthology. 2004;78:237-42.

Malczewski A, Rocki B, Ramisz A, Eckert J. *Echinococcus multilocularis* (Cestoda), the causative agent of alveolar echinococcosis in humans - first record in Poland. Journal of Parasitology. 1995;81(2):318-21.

Malczewski A, Ramisz A, Rocki B, Bienko R, Balicka-Ramisz A, Eckert J. *Echinococcus multilocularis* in red foxes (*Vulpes vulpes*) in Poland: an update of the epidemiological situation. Acta Parasitologica. 1999;44(1):68-72.

Malczewski A, Borecka A, Malczewska M, Gawor J. An attempt to determine intermediate hosts of the tapeworm *Echinococcus multilocularis* in Poland. Wiadomosci parazytologiczne. 2008;54(2):137-41.

Malczewski A, Gawor J, Malczewska M. Infection of red foxes (*Vulpes vulpes*) with *Echinococcus multilocularis* during the years 2001-2004 in Poland. Parasitology Research. 2008;103(3):501-5.

Manfredi MT, Genchi C, Deplazes R, Trevisiol K, Fraquelli C. *Echinococcus multilocularis* infection in red foxes in Italy. Veterinary Record. 2002;150(24):757-.

Manfredi MT, Di Cerbo AR, Trevisiol K. An updating on the epidemiological situation of *Echinococcus multilocularis* in Trentino Alto Adige (northern Italy). Parassitologia. 2004;46(4):431-3.

Manfredi MT, Casulli A, La Rosa G, Di Cerbo AR, Trevisio K, Genchi C, et al. *Echinococcus multilocularis* in north Italy. Parassitologia. 2006;48(1-2):43-6.

Manke KJ, Stoye M. Parasitological studies of red foxes (*Vulpes vulpes* L.) in the northern districts of Schleswig-Holstein. Tieraerztliche Umschau. 1998;53:207-14.

Martinek K, Kolarova L, Cerveny J, Andreas M. *Echinococcus multilocularis* (Cestoda : Taeniidae) in the Czech Republic: the first detection of metacestodes in a naturally infected rodent. Folia Parasitologica. 1998;45(4):332-3.

Martinek K, Kolarova L, Cerveny J. *Echinococcus multilocularis* in carnivores from the Klatovy district of the Czech Republic. Journal of Helminthology. 2001;75(1):61-6.

Mathy A, Hanosset R, Adant S, Losson B. The carriage of larval *Echinococcus multilocularis* and other cestodes by the muskrat (*Ondatra zibethicus*) along the ourthe river and its tributaries (Belgium). Journal of Wildlife Diseases. 2009;45(2):279-87.

Meine K, Muller P. On the occurrence of the small fox tapeworm Echinococcus multilocularis (Leuckart 1863) in the Saarland. Zeitschrift Fur Jagdwissenschaft. 1996;42(4):274-83.

Meyer H, Svilenov D. Finding of *Echinococcus multilocularis* in stray domestic cats in South Germany. Zentralblatt Fur Veterinarmedizin Reihe B-Journal of Veterinary Medicine Series B-Infectious Diseases Immunology Food Hygiene Veterinary Public Health. 1985;32(10):785-6.

Miterpakova M, Varady M, Reiterova K, Turcekova L, Dubinsky P. Present state of the occurrence of *Echinococcus multilocularis* in red foxes in Slovakia. Helminthologia. 2001;38:182.

Miterpakova M, Dubinsky P, Reiterova K, Machkova N, Varady M, Snabel V. Spatial and temporal analysis of the *Echinococcus multilocularis* occurrence in the Slovak Republic. Helminthologia. 2003;40(4):217-26.

Miterpakova M, Dubinsky P, Reiterova K, Stanko M. Climate and environmental factors influencing *Echinococcus multilocularis* occurrence in the Slovak Republic. Annals of Agricultural and Environmental Medicine. 2006;13(2):235-42.

Miterpakova M, Hurnikova Z, Antolova D, Dubinsky P. Endoparasites of red fox (*Vulpes vulpes*) in the Slovak Republic with the emphasis on zoonotic species *Echinococcus multilocularis* and *Trichinella* spp. Helminthologia. 2009;46(2):73-9.

Miterpakova M, Dubinsky P. Fox tapeworm (*Echinococcus multilocularis*) in Slovakia - summarizing the long-term monitoring. Helminthologia. 2011;48(3):155-61.

Moks E, Saarma U, Valdmann H. *Echinococcus multilocularis* in Estonia. Emerging Infectious Diseases. 2005;11(12):1973-4.

Moks E. Tapeworm parasites *Echinococcus multilocularis* and *E. granulosus* in Estonia: phylogenetic relationships and occurrence in wild carnivores and ungulates. PhD Thesis Universitatis Tartuensis. 2008.

Monnier Ph, Cliquet F, Aubert M, Bretagne S, Monnier P. Improvement of a polymerase chain reaction assay for the detection of *Echinococcus multilocularis* DNA in faecal samples of foxes. Veterinary Parasitology. 1996;67(3–4):185–95.

Muehling A, Zeyhle E, Frank W. Epidemiological studies on *Echinococcus multilocularis* in southwest Germany. Proceedings of the second International Symposium on taeniasis/cysticercosis and echinococcosis/hydatidosis 2-7 1985.

Muller B, Partridge A. The occurrence of *Echinococcus multilocularis* in animals in South Wurttemberg. Tierarztliche Umschau. 1974;29:602-12.

Murphy TM, Wahlstrom H, Dold C, Keegan JD, McCann A, Melville J, et al. Freedom from *Echinococcus multilocularis*: An Irish perspective. Veterinary Parasitology. 2012;190(1-2):196-203.

Nagy A, Ziadinov I, Schweiger A, Schnyder M, Deplazes P. Hair coat contamination with zoonotic helminth eggs of farm and pet dogs and foxes. Berlinerund Munchener Tierarztliche Wochenschrift. 2011;124:503-11.

Nicodemus S. Echinococcus multilocularis and other Cestoda larvae in muskrat (*Ondatra zibethicus*) in Luxembourg. PhD Thesis. Universitat Hohenheim. 2012.

Osterman Lind E, Juremalm M, Christensson D, Widgren S, Hallgren G, Agren EO, et al. First detection of *Echinococcus multilocularis* in Sweden, February to March 2011. Eurosurveillance. 2011;16(14):1-3.

Pacon J, Soltysiak Z, Nicpon J, Janczak M. Prevalence of internal helminths in red foxes (*Vulpes vulpes*) in selected regions of Lower Silesia. Medycyna Weterynaryjna. 2006;62(1):67-9.

Pavlasek I, Chalupsky J, Kolarova L. *Echinococcus multilocularis* - a little tapeworm of foxes. Veterinarstvi. 1996;4:164-7.

Pavlasek I, Chalupsky J, Kolarova L, Horyna B, Ritter J. Occurrence of *Echinococcus multilocularis* Leuckart, 1863, in foxes (*Vulpes vulpes*) in the Czech Republic. Epidemiologie, mikrobiologie, imunologie : casopis Spolecnosti pro epidemiologii a mikrobiologii Ceske lekarske spolecnosti JE Purkyne. 1997;46(4):158-62.

Pavlasek I. Actual situation and occurence of *Echinococcus multilocularis* in foxes both in Europe and in Czech Republic. Remedia - Klinicka mikrobiologie. 1998;2:233-40.

Petavy AF, Deblock S. The Auvergnan focus of alveolar echinococcosis. Research on the intermediate host , description of the lesions. Annales de parasitologie humaine et comparee. 1983;58:439-53.

Petavy AF, Deblock S, Gilot B.First occurrence of the larval stage of *Echinococcus multilocularis* in *Microtus arvalis* and *Clethrionomys glareolus* in a focus of alveolar hydatidosis in the Massif Central (France). Comptes Rendus de l’Academie des Sciences, III (Sciences de la Vie). 1984;299(18):735–7.

Petavy AF, Duriez T, Gilot B, Deblock S. Status of the focus of multilocular hydatidiosis in the Auvergne. Fourth year of study. Bulletin de la Societe Francaise de Parasitologie. 1985;1:115-8.

Petavy AF, Deblock S, Prost C. Epidemiology of alveolar echinococcosis in France. 1. Intestinal helminths in the red fox (*Vulpes vulpes* L.) from Haute-Savoie. Annales de parasitologie humaine et comparee. 1990;65:22-7.

Petavy AF, Deblock S, Walbaum S. Life cycles of *Echinococcus multilocularis* in relation to human infection. Journal of Parasitology. 1991;77(1):133-7.

Petavy AF, Tenora F, Deblock S. Contributions to knowledge on the helminths parasitizing several Arvicolidae (Rodentia ) in Auvergne (France). Helminthologia. 1996;33:51-8.

Petavy AF, Tenora F, Deblock S, Sergent V. *Echinococcus multilocularis* in domestic cats in France. A potential risk factor for alveolar hydatid disease contamination in humans. Veterinary Parasitology. 2000;87(2-3):151-6.

Petavy AF, Tenora F, Deblock S. Co-occurrence of metacestodes of *Echinococcus multilocularis* and *Taenia taeniaeformis* (Cestoda) in *Arvicola terrestris* (Rodentia) in France. Folia Parasitologica. 2003;50(2):157-8.

Pesson B, Carbiener R. Ecology of multilocular hydatidosis in Alsace. Parasitism in the red fox (*Vulpes vulpes*). Bulletin d'Ecologie. 1989;20:295-301.

Pfeiffer AS, Boeckeler W, Lucius R. Parasites of the domestic and wild animals of Schleswig-Holstein West Germany parasites of the inner organs of the Beech Marten (*Martes foina*). Zeitschrift fuer Jagdwissenschaft. 1989;35(2):100–12.

Pfeiffer F, Kuschfeldt S, Stoye M. The helminth fauna of the red fox (Vulpes vulpes Linne 1758) in the south of Saxe-Anhalt .1. Cestodes. Deutsche Tierarztliche Wochenschrift. 1997;104:445-8.

Prosl H, Schmid E. Prevalence of *Echinococcus multilocularis* in foxes in Vorarlberg, Austria. Mitteilungen der Oesterreichischen Gesellschaft fuer Tropenmedizin und Parasitologie. 1991;13:41-6.

Ramisz A, Eckert J, BalickaRamisz A, Grupinski T, Pilarczyk B, KrolPospieszny A, et al. Prevalence of *Echinococcus multilocularis* in foxes in the Western Poland. Medycyna Weterynaryjna. 1997;53(6):340-2.

Ramisz A, Eckert J, Balicka-Ramisz A, Bienko R, Pilarczyk B. Epidemiological studies on *Echinococcus multilocularis* in red foxes in north-west Poland. Wiadomosci parazytologiczne. 1999;45(3):369-73.

Ramisz A, Nicpon J, Balicka-Ramisz A, Pilarczyk B, Pacon J, Piekarska J. The prevalence of gastro-intestinal helminths in red foxes (*Vulpes vulpes*) in the south-west part of Poland. Tierarztliche Umschau. 2004;59(10):601-4.

Raoul F, Deplazes P, Nonaka N, Piarroux R, Vuitton DA, Giraudoux P. Assessment of the epidemiological status of *Echinococcus multilocularis* in foxes in France using ELISA coprotests on fox faeces collected in the field. International Journal for Parasitology. 2001;31(14):1579-88.

Rehmann P, Grone A, Gottstein B, Sager H, Muller N, Vollm J, Bacciarini LN. Alveolar echinococcosis in the zoological garden Basle. Schweizer Archiv fur Tierheilkunde. 2005;147(11):498–502.

Reiterova K, Miterpakova M, Turckova U, Antolova D, Dubinsky P. Field evaluation of an intravital diagnostic test of *Echinococcus multilocularis* infection in red foxes. Veterinary Parasitology. 2005;128(1-2):65-71.

Reiterova K, Dziemian E, Miterpakova M, Antolova D, Kolodziej-Sobocinska M, Machnicka B, et al. Occurrence of *Echinococcus multilocularis* in red foxes from the Carpathian regions of Slovakia and Poland. Acta Parasitologica. 2006;51(2):107-10.

Remde I. Investigations on the occurrence of *Echinococcus multilocularis* and *Trichinella* spp. in wild boars (*Sus scrofa scrofa*) in the Wartburg region. Freie Universitaet Berlin, Berlin DT Dissertation. 2008;110 p.

Reperant LA, Weber JM, Hegglin D, Deplazes P. *Echinococcus multilocularis* infections of rural, residential and urban foxes (*Vulpes vulpes*) in the canton of Geneva, Switzerland. Parasite-Journal De La Societe Francaise De Parasitologie. 2005;12(4):339-46.

Reperant Leslie A, Hegglin D, Fischer C, Kohler L, Weber J-M, Deplazes P. Influence of urbanization on the epidemiology of intestinal helminths of the red fox (*Vulpes vulpes*) in Geneva, Switzerland. Parasitology research. 2007;101:605-11.

Reperant LA, Hegglin D, Tanner I, Fischer C, Deplazes P. Rodents as shared indicators for zoonotic parasites of carnivores in urban environments. Parasitology. 2009;136:329-37.

Robardet E, Giraudoux P, Caillot C, Boue F, Cliquet F, Augot D, et al. Infection of foxes by *Echinococcocus multilocularis* in urban and suburban areas of Nancy, France: influence of feeding habits and environment. Parasite (Paris, France) 2008;15:77-85.

Rocki B, Malczewski A, Eckert J. Studies on the incidence of *Echinococcus multilocularis* in red foxes (*Vulpes vulpes*) in north-east, central and south of Poland. Wiadomosci parazytologiczne. 1999;45(3):391-3.

Romig T, Bilger B, Dinkel A, Merli M, Thoma D, Will R, et al. Impact of praziquantel baiting on intestinal helminths of foxes in southwestern Germany. Helminthologia. 2007;44:137-44.

Saeed I, Maddox-Hyttel C, Monrad J, Kapel CMO. Helminths of red foxes (*Vulpes vulpes*) in Denmark. Veterinary Parasitology. 2006;139:168-79.

Saegerman C, Blander Hd, Hanosset R, Berkvens D, Losson B, Brochier B, et al. Risk assessment of the presence of *Echinococcus multilocularis* and *Toxocara canis* in foxes from Brussels. Epidemiologie et Sante Animale. 2006;50:97-104.

Sager H, Moret CS, Grimm F, Deplazes P, Doherr MG, Gottstein B. Coprological study on intestinal helminths in Swiss dogs: temporal aspects of anthelminthic treatment. Parasitology Research. 2006;98(4):333-8.

Schelling U, Schafer E, Pfister T, Frank W. An epidemiologic-study of the prevalence of *Echinococcus multilocularis* in north-east Baden-Wurttemberg. Tierarztliche Umschau. 1991;46(11):673-6.

Schelling U, Frank W, Will R, Romig T, Lucius R. Chemotherapy with praziquantel has the potential to reduce the prevalence of *Echinococcus multilocularis* in wild foxes (*Vulpes vulpes*). Annals of Tropical Medicine and Parasitology. 1997;91(2):179-86.

Schichowski HD. Investigations on the occurrence of finned stadia of *Echinococcus multilocularis* in muskrats (*Ondatra zibethicus*) in the district of Arnsberg North-Rhine Westfalia. Zeitschrift Fur Jagdwissenschaft. 2002;48(2):119-24.

Schmitt M, Saucy F, Wyborn S, Gottstein B. Infestation of water voles (*Arvicola terrestris*) with metacestodes of *Echinococcus multilocularis* in the canton of Freiburg (Switzerland). Schweizer Archiv Fur Tierheilkunde. 1997;139(2):84-93.

Schoffel I, Schein E, Wittstadt U, Hentsche J. Parasite fauna of red foxes in Berlin (West). Berliner und Munchener tierarztliche Wochenschrift. 1991;104(5):153–7.

Schott E, Muller B. Prevalence of *Echinococcus multilocularis* in foxes in the district of Tubingen, West-Germany. Tierarztliche Umschau. 1989;44(6):367-70.

Schott E, Muller B. Age specific prevalences of *Echinococcus multilocularis* infection in red foxes (*Vulpes vulpes*). Tierarztliche Umschau. 1990;45(9):620-3.

Schwarz S, Sutor A, Staubach C, Mattis R, Tackmann K, Conraths FJ. Estimated prevalence of *Echinococcus multilocularis* in raccoon dogs *Nyctereutes procyonoides* in northern Brandenburg, Germany. Current Zoology. 2011;57(5):655-61.

Seegers G, Baumeister S, Pohlmeyer K, Stoye M. *Echinococcus multilocularis* -metacestodes in muskrats (*Ondatra zibethicus)* in Lower Saxony. Deutsche Tieraerztliche Wochenschrift. 1995;102(6):256.

Siko Barabasi S, Bokor E, Fekeas E, Nemes I, Murai E, Gubanyi A, et al. Occurrence and epidemiology of *Echinococcus granulosus* and *E. multilocularis* in the Covasna County, East Carpathian Mountains, Romania. Parasitologia Hungarica. 1995;28:43-56.

Siko Barabasi S, Deplazes P, Cozma V, Pop S, Tivadar C, Bogolin I, et al. *Echinococcus multilocularis* confirmed in Romania. . Scientia Parasitologica. 2010;11:89-96.

Siko Barabasi S, Fok E, Gubanyi A, Meszaros F, Cozma V. Helminth fauna of the small intestine in the European red fox , *Vulpes vulpes* with notes on the morphological identification of *Echinococcus multilocularis.* Scientia Parasitologica. 2010;11:141-51.

Siko Barabasi S, Marosfoi L, Siko Barabasi Z, Cozma V. Natural alveolar echinococcosis with *Echinococcus multilocularis* in wild rodents. Scientia Parasitologica. 2011;12 (1):11–21.

Smith GC, Gangadharan B, Taylor Z, Laurenson MK, Bradshaw H, Hide G, et al. Prevalence of zoonotic important parasites in the red fox (*Vulpes vulpes*) in Great Britain. Veterinary parasitology. 2003;118:133-42.

Staubach C, Thulke HH, Tackmann K, Hugh-Jones M, Conraths FJ. Geographic information system-aided analysis of factors associated with the spatial distribution of *Echinococcus multilocularis* infections of foxes. American Journal of Tropical Medicine and Hygiene. 2001;65(6):943-8.

Sreter T, Szell Z, Egyed Z, Varga I. *Echinococcus multilocularis*: An emerging pathogen in Hungary and Central Eastern Europe? Emerging Infectious Diseases. 2003;9(3):384-6.

Sreter T, Szell Z, Sreter-Lancz Z, Varga I. *Echinococcus multilocularis* in northern Hungary. Emerging Infectious Diseases. 2004;10(7):1344-6.

Staubach C, Hoffmann L, Schmid VJ, Ziller M, Tackmann K, Conraths FJ. Bayesian space-time analysis of *Echinococcus multilocularis* infections in foxes. Veterinary Parasitology. 2011;179(1-3):77-83.

Stieger C, Hegglin D, Schwarzenbach G, Mathis A, Deplazes P. Spatial and temporal aspects of urban transmission of *Echinococcus multilocularis*. Parasitology. 2002;124:631-40.

Stien A, L. V, Haukisalmi V, Fuglei E, Mork T, Yoccoz NG, et al. Intestinal parasites of the Arctic fox in relation to the abundance and distribution of intermediate hosts. Parasitology. 2010;137:149-57.

Suhrke J, Plotner J, Zemke M. Occurrence of *Echinococcus multilocularis* in animals in Southern Thuringia. Monatshefte Fur Veterinarmedizin. 1991;46(20):714-7.

Sydler T, Mathis A, Deplazes P. *Echinococcus multilocularis* lesions in the livers of pigs kept outdoors in Switzerland. European Journal of Veterinary Pathology. 1998;4(1):43–6.

Szabova E, Juris P, Miterpakova M, Antolova D, Papajova I, Sefcikova H. Prevalence of important zoonotic parasites in dog populations from the Slovak Republic. Helminthologia 2007;44:170-6.

Szell Z, Marucci G, Pozio E, Sreter T. *Echinococcus multilocularis* and *Trichinella spiralis* in golden jackals (*Canis aureus*) of Hungary. Veterinary Parasitology. 2013;197(1-2):393-6.

Tackmann K, Beier D. The prevalence of *Echinococcus multilocularis* infection in wildlife carnivores in an area of Germany .1. Parasitological analysis of wild carnivores for determination of pathogen prevalence. Tierarztliche Umschau. 1993;48(8):498-503.

Tackmann K, Loschner U, Mix H, Staubach C, Thulke HH, Conraths FJ. Spatial distribution patterns of *Echinococcus multilocularis* (Leuckart 1863) (Cestoda: Cyclophyllidea: Taeniidae) among red foxes in an endemic focus in Brandenburg, Germany. Epidemiology and infection. 1998;120(1):101–9.

Tackmann K, Loschner U, Mix H, Staubach C, Thulke HH, Ziller M, et al. A field study to control *Echinococcus multilocularis* infections of the red fox (*Vulpes vulpes*) in an endemic focus. Epidemiology and Infection. 2001;127(3):577-87.

Takumi K, de Vries A, Chu ML, Mulder J, Teunis P, van der Giessen J. Evidence for an increasing presence of *Echinococcus multilocularis* in foxes in The Netherlands. International Journal for Parasitology. 2008;38(5):571-8.

Tanner F, Hegglin D, Thoma R, Brosi G, Deplazes P. *Echinococcus multilocularis* in Grisons: distribution in foxes and presence of potential intermediate hosts. Schweizer Archiv Fur Tierheilkunde. 2006;148(9):501-10.

Teysseyre A. Contribution to the study of internal parasitism of foxes (*Vulpes vulpes*). Thesis, University of Toulouse. 2005.

Thiess A, Schuster R, Nockler K, Mix H. Helminth findings in indigenous raccoon dogs (*Nyctereutes procyonoides)* (Grey, 1843). Berliner und Munchener tierarztliche Wochenschrift. 2001;114(7–8):273–6.

Thiess A. Studies on the helminth fauna and the occurrence of *Trichinella* species of the raccoon dog (*Nyctereutes procyonoides*) in the Federal State Brandenburg. PhD Thesis. Mensch & Buch Verlag, Berlin. 2004.

Tolnai Z, Szell Z, Sreter T. Environmental determinants of the spatial distribution of *Echinococcus multilocularis* in Hungary. Veterinary Parasitology. 2013;198(3-4):292-7.

Uhl W, Betke P, Decker J. Postmortem findings in red foxes. Praktische Tierarzt. 1993;74:1018-24.

Umhang G, Woronoff-Rhen N, Combes B, Boue F. Segmental Sedimentation and Counting Technique (SSCT): An adaptable method for qualitative diagnosis of *Echinococcus multilocularis* in fox intestines. Experimental Parasitology. 2011;128(1):57-60.

Umhang G, Raton V, Comte S, Hormaz V, Boucher J-M, Combes B, et al. *Echinococcus multilocularis* in dogs from two French endemic areas: No evidence of infection but hazardous deworming practices. Veterinary Parasitology. 2012;188(3-4):301-5.

Umhang G, Richomme C, Boucher J-M, Guedon G, Boue F. Nutrias and Muskrats as bioindicators for the presence of *Echinococcus multilocularis* in new endemic areas. Veterinary Parasitology. 2013;197(1-2):283-7.

Umhang G, Comte S, Raton V, Hormaz V, Boucher J-M, Favier S, et al. *Echinococcus multilocularis* infections in dogs from urban and peri-urban areas in France. Parasitology Research. 2014;113(6):2219-22.

van der Giessen JWB, Rombout YB, Franchimont JH, Limper LP, Homan WL. Detection of *Echinococcus multilocularis* in foxes in the Netherlands. Veterinary Parasitology. 1999;82(1):49-57.

van der Giessen JWB, Rombout YB, Evers EG. Base line prevalence of *Echinococcus multilocularis* in foxes in the Netherlands. Acta Parasitologica. 2000;45(3):238pp.

van der Giessen JWB, Rombout Y, Teunis P. Base line prevalence and spatial distribution of *Echinococcus multilocularis* in a newly recognized endemic area in the Netherlands. Veterinary Parasitology. 2004;119(1):27-35.

van der Giessen J, Vervaeke M, de Vries A, Chu M, Brochier L, Losson B, et al. Is *Echinococcus multilocularis* increasing in prevalence in the Western European border line? International Journal of Antimicrobial Agents. 2007;29:S51-S.

van Gucht S, Van Den Berge K, Quataert P, Verschelde P, Le Roux I. No Emergence of *Echinococcus multilocularis* in foxes in Flanders and Brussels Anno 2007-2008. Zoonoses and Public Health. 2010;57(7-8):E65-E70.

Vergles Rataj A, Bidovec A, Zele D, Vengust G. *Echinococcus multilocularis* in the red fox (*Vulpes vulpes*) in Slovenia. European Journal of Wildlife Research. 2010;56(5):819-22.

Vergles Rataj A, Posedi Janez ZD, Vengust G. Intestinal parasites of the red fox (V*ulpes vulpes*) in Slovenia. 2013;61(4):1-9.

Vervaeke M, Dorny P, Vercammen F, Geerts S, Brandt J, Van Den Berge K, Verhagen R. *Echinococcus multilocularis* (Cestoda, Taeniidae) in red foxes (*Vulpes vulpes*) in northern Belgium. Veterinary parasitology. 2003;115(3):257–63.

Vervaeke M, Dorny P, De Bruyn L, Vercammen F, Jordaens K, Van Den Berge K, Verhagen R. A survey of intestinal helminths of red foxes (*Vulpes vulpes*) in northern Belgium. Acta Parasitologica. 2005;50(3):221–7.

Vervaeke M, van der Giessen J, Brochier B, Losson B, Jordaens K, Verhagen R, et al. Spatial spreading of *Echinococcus multilocularis* in red foxes (*Vulpes vulpes*) across nation borders in Western Europe. Preventive Veterinary Medicine. 2006;76(3-4):137-50.

von Keyserlingk M, Thoms B, Korfer KH, Braune S. Investigations of the occurrence of *Echinococcus multilocularis* in the red fox population of Lower Saxony. Tierarztliche Umschau. 1998;53(4):202-207.

Vos A, Schneider L. The prevalence of *Echinococcus multilocularis* in red foxes (*Vulpes vulpes*) in Southern Bavaria. Tierarztliche Umschau. 1994;49(4):225.

Wahlstrom H, Isomursu M, Hallgren G, Christensson D, Cedersmyg M, Wallensten A, et al. Combining information from surveys of several species to estimate the probability of freedom from *Echinococcus multilocularis* in Sweden, Finland and mainland Norway. Acta Veterinaria Scandinavica. 2011;53.

Wahlstrom H, Lindberg A, Lindh J, Wallensten A, Lindqvist R, Plym-Forshell L, et al. Investigations and actions taken during 2011 due to the first finding of *Echinococcus multilocularis* in Sweden. Eurosurveillance. 2012;17(28):10-7.

Wahlstrom H, Botero-Kleiven S, Lind EO, Christensson D, Cedersmyg M, Agren EO. Present status of *Echinococcus multilocularis* in Sweden. Tropical Medicine & International Health. 2013;18:96-7.

Welzel AM, Steinbach G. von Keyserlingk M, Stoye M, Von Keyserlingk M. On the helminth fauna of red foxes (*Vulpes vulpes* L.) in southern Lower Saxony. Part 2: cestodes. Zeitschrift fuer Jagdwissenschaft. 1995; 41(2):100–109.

Wessbecher H, Dalchow W, Stoye M. The helminth fauna of the red fox (*Vulpes vulpes* Linne 1758) in the administrative district of Karlsruhe. 1. Cestodes. Deutsche tierarztliche Wochenschrift. 1994; 101: 322-6.

Worbes H. The occurrence of *Echinococcus granulosus and E. multilocularis* in Thuringia. Angewandte Parasitologie. 1992;33(4):193-204.

Zeyhle E. *Echinococcus multilocularis* in fox (*Vulpes vulpes*), field voles ( *Microtus arvalis*) and humans in an endemic area of the Schwabische Alb. Praktische Tierarzt. 1980;61(4):360.

Zeyhle E, Abel M, Frank W. Epidemiological studies on the occurrence of *Echinococcus multilocularis* in definitive and intermediate hosts in Germany. Mitteilungen der Oesterreichischen Gesellschaft fuer Tropenmedizin und Parasitologie. 1990; 12:221-32.
