# Supplementary material for: The geographical distribution and prevalence of Echinococcus multilocularis in animals in the European Union and adjacent countries: a systematic review and meta-analysis
Source: Parasit Vectors. 2016 Sep 28;9:519. doi: 10.1186/s13071-016-1746-4 (PMC5039905; doi:10.1186/s13071-016-1746-4)
Supplement: Additional file 4: Text S4. — List of excluded studies. (DOCX 45 kb) [file 13071_2016_1746_MOESM4_ESM.docx]

**S4 Text: List of excluded studies**

| REFERENCE | REASON for EXCLUSION |
| --- | --- |
| Artois, M.; Stahl, P.; Bonnin, J.L.; et. al. Hosts of *E.multilocularis* in Lorraine and their consequences on human contamination. II. Analytical study: Fox - rodents relationship and occurence of the parasite. Bulletin de la Societe Francaise de Parasitologie. 1986;4(1):65–72. | Not a primary research |
| Balicka-Ramisz, A.; Ramisz, A.; Pilarczyk, B.; Bienko, R. Fauna of gastro-intestinal parasites in red foxes in Western Poland. Medycyna Weterynaryjna. 2003;59(10):922–5. | Not a primary research |
| Berke, O. Choropleth mapping of regional count data of *Echinococcus multilocularis* among red foxes in Lower Saxony, Germany. Preventative veterinary medicine. 2001;52(2):119–31. | Not a primary research |
| Bessonov, A. S. *Echinococcus multilocularis* infection in Russia and neighbouring countries. Helminthologia. 1998;35(2):73–8. | Not a primary research |
| Bessonov, A. S. Echinococcoses of animals and humans in the Russian Federation. NATO Science Series: Life and Behavioural Sciences. 2002;341:91–8. | Not a primary research |
| Brossard, M. Aspects of the cycle of *Echinococcus multilocularis* and of the epidemiology of alveolar echinococcosis in the Jura mountains of Switzerland. Revue medicale de la Suisse romande. 1989;109(2):85–7. | Not a primary research |
| Carmena D.; Cardona G. A. Canine echinococcosis: Global epidemiology and genotypic diversity. [Acta Trop.](http://www.ncbi.nlm.nih.gov/pubmed/?term=Canine+echinococcosis+%3A+Global+epidemiology+and+genotypic+diversity##) 2013;128(3):441–60. | Not a primary research |
| Chermette, R. Importance and current status of larval echinococcosis in France. II. *Echinococcus multilocularis*. Point Veterinaire. 1983;15(74):29–35. | Not a primary research |
| Colas, F.; Deiller, M. Echinococcosis due to *Echinococcus multilocularis* in France: life cycle. Epidemiologie et Sante Animale. 1987;12:107–23. | Not a primary research |
| Contat, F.; Petavy, A.F.; Deblock, S.; Euzeby, J. Epidemiological study of two foci of alveolar hydatidosis in France. Sciences Veterinaires Medecine Comparee. 1983;85(2):79–82. | Not a primary research |
| Craig, P. *Echinococcus multilocularis*. Current opinion in infectious diseases. 2003;16(5):437–44. | Not a primary research |
| Davidson R. K.; Romig T.; Jenkins E.; Tryland M.; Robertson L.J. The impact of globalisation on the distribution of *Echinococcus multilocularis*. [Trends Parasitol.](http://www.ncbi.nlm.nih.gov/pubmed/?term=The+impact+of+globalisation+on+the+distribution+of+Echinococcus++multilocularis##) 2012; 28(6):239–47. | Not a primary research |
| Deblock, S.; Petavy, A.F. Recent data on the epidemiology of alveolar hydatid disease in France. Bulletin de la Societe de Pathologie Exotique et de ses Filiales. 1990;83(2):242–48. | Not a primary research |
| Deplazes, P.; Eckert, J. Veterinary aspects of alveolar echinococcosis - a zoonosis of public health significance. Veterinary parasitology. 2001;98(1–3):65–87. | Not a primary research |
| Deplazes, P.; Schweiger, A.; van Knapen, F.; Overgaauw, P.A.M. Role of pet dogs and cats in the transmission of helminthic zoonoses in Europe, with a focus on echinococcosis and toxocarosis. [Vet Parasitol.](http://www.ncbi.nlm.nih.gov/pubmed/?term=Role+of+pet+dogs+and+cats+in+the+transmission+of+helminthic+zoonoses+in+Europe%2C+with+a+focus+on+echinococcosis+and+toxocarosis.##) 2011;182(1):41–53. | Not a primary research |
| Deplazes P. Ecology and epidemiology of *Echinococcus multilocularis* in Europe. Parassitologia. 2006;48(1–2):37–9. | Not a primary research |
| Duscher, G.; Pleydell, D.; Prosl, H.; Joachim, A. *Echinococcus multilocularis* in Austrian foxes from 1991 until 2004. Journal of veterinary medicine. B, Infectious diseases and veterinary public health. 2006;53(3):138–44. | Not a primary research |
| Eckert, J. The “dangerous fox tapeworm” (*Echinococcus multilocularis*) and alveolar echinococcosis of humans in central Europe. Berliner und Munchener tierarztliche Wochenschrift. 1996;109(6–7):202–10. | Not a primary research |
| Eckert, J. Epidemiology of *Echinococcus multilocularis* and *E. granulosus* in central Europe. Parassitologia. 1997;39(4):337–44. | Not a primary research |
| Eckert, J.; Deplazes, P. Actual aspects in the epidemiology of echinococcosis in Europe. ISS/WHO/FAO-CC/IZSTe/98.29 Modern methods for the diagnosis and control of echinococcosis in dogs. Veterinary public health reports. 1999:81–95. | Not a primary research |
| Eckert, J.; Conraths, F.J.; Tackmann, K. Echinococcosis : an emerging or re-emerging zoonosis? International journal for parasitology. 2000;30(12–13):1283–94. | Not a primary research |
| Eckert, J.; Gemmell, M.A.; Meslin, F.X.; Pawowski, Z.S. WHO/OIE manual on echinococcosis in humans and animals: a public health problem of global concern. 2001: pp. i-xvii + 265 pp | Not a primary research |
| Eckert, J.; Deplazes, P. Biological, epidemiological, and clinical aspects of echinococcosis, a zoonosis of increasing concern. Clinical microbiology reviews. 2004;(1):107–35. | Not a primary research |
| Fessler, M. Comparison of endemic districts for *Echinococcus multilocularis* and for rabies in Central Europe. Schweizer Archiv fuer Tierheilkunde. 1991;133(4):187pp. Abstract of dissertation, Vet. Med. Fac., Zurich, 1990. | Not a primary research |
| Frank, W. *Echinococcus multilocularis* an endemic cestode of foxes in south Germany, biology, epidemiology , and public-health importance. Wiener Tierarztliche Monatsschrift. 1984;71(1):19–22. | Not a primary research |
| Gawor, J.; Malczewski, A.; Stefaniak, J.; Nahorski, W.; Paul, M.; Kacprzak, E.; Myjak, P. Risk of alveococcosis for humans in Poland. Przeglad epidemiologiczny. 2004;58(3):459–65. | Not a primary research |
| Gawor, J.; Malczewski, A.; Rocki, B. *Echinococcus multilocularis* tapeworm in red foxes (*Vulpes vulpes*) and their danger for humans. Medycyna Weterynaryjna. 2004; 60(4):349–51. | Not a primary research |
| Gawor, J.; Malczewski, A.; Rocki, B.; Malczewska, M.; Borecka, A. Prevalence of the dangerous for humans tapeworm *Echinococcus multilocularis* in red foxes in Poland. Medycyna Weterynaryjna. 2004;60(5):489–91. | Not a primary research |
| Gawor, J.; Borecka, J.; Malczewski, A. The infection of red foxes with *Echinococcus multilocularis* as potential risk for humans in Poland. Zycie Weterynaryjne. 2008;83(1):24–7. | Not a primary research |
| Gilot, B.; Petavy, A.F.; Contat, F. Alveolar hydatidosis: epidemiological review. Epidemiologie et Sante Animale. 1983;4:17–28 | Not a primary research |
| Giraudoux, P.; Raoul, F.; Bardonnet, K.; Vuillaume, P.; Tourneux, F.; Cliquet, F.; Delattre, P.; Vuitton, D.A. Alveolar echinococcosis: characteristics of a possible emergence and new perspectives in epidemiosurveillance. Medecine et Maladies Infectieuses. 2001;31(S2):247S-56S. | Not a primary research |
| Giraudoux, P.; Raoul, F.; Boue, F.; Combes, B.; Piarroux, R.; Bresson-Hadni, S.; Vuitton, D.-A. Geography of alveolar echinococcosis. Bulletin de l’Academie nationale de medicine. 2008;192(6):1119–25. | Not a primary research |
| Guillot, J.; Bouree, P. Helminths transmissible from domestic carnivores to man: risk assessment and prevention strategies. Bulletin de l’Academie Nationale de Medecine. 2007;191(1):67–81. | Not a primary research |
| Hanosset, R.; Mignon, B.; Losson, B. Recent data on a current zoonosis: alveolar echinococcus due to *Echinococcus multilocularis*. Annales de Medecine Veterinaire. 2004;148(4):153–67. | Not a primary research |
| Kantere, M.; Athanasiou, L.V.; Chatzopoulos, D.C.; Spyrou, V.; Valiakos, G.; Kontos, V.; Billinis, C. Enteric pathogens of dogs and cats with public health implications. American Journal of Animal and Veterinary Sciences. 2014;9(2):84–94. | Not a primary research |
| Kolarova, L. *Echinococcus multilocularis*: new epidemiological insights in Central and Eastern Europe. Helminthologia. 1999;36(3):193–200. | Not a primary research |
| Kosmider, R.; Paterson, A.; Voas, A.; Roberts, H. *Echinococcus multilocularis* introduction and establishment in wildlife via imported beavers. The Veterinary record. 2013;172(23):606pp. | Not a primary research |
| Le Pesteur, M.H.; Giraudoux, P.; Delattre, P.; Damange, J.P.; Quere, J.P. Spatiotemporal distribution of four species of cestodes in a landscape of mid-altitude mountains (Jura, France). Annales de Parasitologie Humaine et Comparee. 1992;67(5):155–60. | Not a primary research |
| Letkova, V. Helminthozoonoses the current situation in Slovakia. Folia Veterinaria. 2006;50(4):201–4. | Not a primary research |
| Lewis Fraser, I.; Otero-Abad, B.; Torgerson, P.R; Hegglin, D.; Deplazes, P. Dynamics of the force of infection: insights from *Echinococcus multilocularis* infection in foxes. PLoS neglected tropical diseases. 2014;8(3):e2731. | Not a primary research |
| Lucius, R.; Bilger, B. *Echinococcus multilocularis* in Germany: increased awareness or spreading of a parasite? Parasitology today. 1995;11(11):430–4. | Not a primary research |
| Lukashenko, N.P. Problems of epidemiology and prophylaxis of alveococosis (multilocular echinococcosis): a general review-with particular reference to the U.S.S.R. International Journal for Parasitology. 1971;1(2):125–34. | Not a primary research |
| Malczewski, A. CE and AE in Eastern Europe. NATO Science Series: Life and Behavioural Sciences, Volume 341. Proceedings of the NATO Advanced Research Workshop on cestode zoonoses: echinococcosis and cysticercosis: an emergent and global problem, Poznan, Poland. 2002:81–9. | Not a primary research |
| Mitro, S. Zoonoses in countries of the former Soviet Union. Osteuropastudien der Hochschulen des Landes Hessen. Reihe 1, Giessener Abhandlungen zur Agrar- und Wirtschaftsforschung des Europaeischen Ostens. 1994;204:134 p. | Not a primary research |
| Mulder, J.L. The raccoon dog (*Nyctereutes procyonoides*) in the Netherlands-its present status and a risk assessment. Lutra. 2013;56(1):23–43. | Not a primary research |
| Okulewicz A.; Hildebrand J.; Okulewicz J.; Perec A. Red fox (*Vulpes vulpes*) as reservoir of parasites and source of zoonosis. [Wiad Parazytol.](http://www.ncbi.nlm.nih.gov/pubmed/16838621##) 2005; 51(2):125–32. | Not a primary research |
| Otero-Abad, B.; Torgerson, P.R. A Systematic Review of the Epidemiology of Echinococcosis in Domestic and Wild Animals. PloS Neglected Tropical Diseases. 2013;7(6):1–16. | Not a primary research |
| Perec-Matysiak, A.; Okulewicz, A.; Hildebrand, J.; Zalesny, G. Helminth parasites of mammals in zoological gardens. Wiadomosci Parazytologiczne. 2007;53(1):15–20. | Not a primary research |
| Petavy, A.F.; Rey, M.; Deblock, S.; Cambon, M. The area of Auvergne, France, a new focus of alveolar echinococcosis. Lyon Medical. 1981;245(S10):111–5. | Not a primary research |
| Petavy A.F.; Deblock S.; Walbaum S. Epidemiology and prevention of echinococcosis in France. La Revue du praticien. 1990;40. | Not a primary research |
| Pfeifer, F. On the occurrence of *Echinococcus multilocularis* and other helminths of the red fox (*Vulpes vulpes* L.) in south Saxony-Anhalt. Tieraerztliche Hochschule Hannover, Hannover DT Dissertation. 1996:151 pp. | Not a primary research |
| Report of a Joint FAO/WHO Expert Meeting. Multi criteria-based ranking for risk management of food-born parasites. Microbiological Risk Assessment Series. 2014;23:xvii + 302 p. | Not a primary research |
| Romig, T.; Bilger, B.; Dinkel, A.; Merli, M.; Mackenstedt, U. *Echinococcus multilocularis* in animal hosts : New data from western Europe. Helminthologia. 1999;36(3):185–91. | Not a primary research |
| Romig, T.; Bilger, B.; Mackenstedt, U. Current spread and epidemiology of *Echinococcus multilocularis*. Deutsche Tierarztliche Wochenschrift. 1999;106(8):352–7. | Not a primary research |
| Romig, T. Spread of *Echinococcus multilocularis* in Europe? NATO Science Series: Life and Behavioural Sciences, Volume 341. Proceedings of the NATO Advanced Research Workshop on cestode zoonoses: echinococcosis and cysticercosis: an emergent and global problem. 2002:65–80. | Not a primary research |
| Romig, T. Epidemiology of echinococcosis. Langenbeck’s archives of surgery /Deutsche Gesellschaft fur Chirurgie. 2003;388(4):209–17. | Not a primary research |
| Romig, T.; Dinkel, A.; Mackenstedt, U. The present situation of echinococcosis in Europe. Parasitology international. 2006;55:S187–91. | Not a primary research |
| Romig, T. *Echinococcus multilocularis* in Europe-state of the art. Veterinary research communications. 2009;33(S1):31–4. | Not a primary research |
| Siko Sandor, B.; Deplazes, P.; Ceica, C.; Tivadar, C.S.; Bogolin, I.; Popescu, S.; Cozma, V. *Echinococcus multilocularis* in south-eastern Europe (Romania). Parasitology research. 2011;108(5):1093–7. | Not a primary research |
| Sutor, A.; Schwarz, S.; Conraths, F.J. The raccoon dog (*Nyctereutes procyonoides*) in Germany - an established Neozoon as host and vector for parasites and other pathogenes. Berliner Munchener Tierarztliche Wochenschrift. 2011;124(11–12):457–64. | Not a primary research |
| Sutor, A.; Schwarz, S.; Conraths, F.J. The biological potential of the raccoon dog (*Nyctereutes procyonoides*, Grey 1834) as an invasive species in Europe - new risks for disease spread? Acta Theriologica. 2014;59(1):49–59. | Not a primary research |
| Torgerson, P.R.; Budke, C.M. Echinococcosis -an international public health challenge. Research in veterinary science. 2003;74(3):191–202. | Not a primary research |
| Umhang, G.; Hormaz, V.; Boue, F.; Knapp, J.; Raoul, F. Using the genetics of *Echinococcus multilocularis* to trace the history of expansion from an endemic area. Infection, Genetics and Evolution. 2014;22:142–9. | Not a primary research |
| van der Giessen, J.W.B.; Borgsteede, F.H.M. *Echinococcus multilocularis* : the prevalence of the fox tapeworm with possible serious consequences for humans. Tijdschrift voor diergeneeskunde. 2002;127(10):318–21. | Not a primary research |
| Vuitton, D.A.; Zhou, H.; Bresson-Hadni, S.; Wang, Q.; Piarroux, M.; Raoul, F.; Giraudoux, P. Epidemiology of alveolar echinococcosis with particular reference to China and Europe. Parasitology. 2003;127:S87–107. | Not a primary research |
| Vuitton, D.A.; Bresson-Hadni, S.; Giraudoux, P.; Bartholomot, B.; Laplante, J.-J.; Delabrousse, E.; Blagosklonov, O.; Mantion, G. Alveolar echinococcosis: from an incurable rural disease to a controlled urban infection. Presse medicale. 2010;39(2):216–30. | Not a primary research |
| Vuitton, D.A.; Wang, Q.; Zhou, H.-X.; Raou,l F.; Knapp, J.; Bresson-Hadni, S.; Wen, H.; Giraudoux, P. A historical view of alveolar echinococcosis , 160 years after the discovery of the first case in humans: part 1. What have we learnt on the distribution of the disease and on its parasitic agent? Chinese medical journal. 2011;124(18):2943–53. | Not a primary research |
| Wahlstrom, H.; Hallgren, G.; Christensson, D.; Uhlhorn, H.; Isomursu, M.; Cedersmyg, M.; Wallensten, A.; Hjertqvist, M.; Davidson, R.K.; Hopp, P. Combining information from surveys of several species to estimate the probability of freedom from *Echinococcus multilocularis* in Sweden, Finland and mainland Norway. Acta Veterinaria Scandinavica. 2011;53:1–13. | Not a primary research |
| Bacciarini, L.N.; Gottstein, B.; Pagan, O.; Rehmann, P.; Grone, A. Hepatic alveolar echinococcosis in cynomolgus monkeys (*Macaca fascicularis*). Veterinary pathology. 2004;41(3):229–34. | Case report |
| Barlow, A.M.; Gottstein, B.; Mueller, N. *Echinococcus multilocularis* in an imported captive European beaver (*Castor fibre*) in Great Britain. The Veterinary record. 2011;169(13):339. | Case report |
| Behrens, S.; Heckers, K.O.; Aupperle, H. Alveolar echinococcosis in an American bulldog in Saxony-Anhalt: A case report. Tieraerztliche Umschau. 2012;67(4):124–8. | Case report |
| Blankenburg, A.; Sauermann, U.; Kaup, F.-J. Spontaneous Echinococcosis in a colony of lion-tailed macaques (*Macaca silenus*). Folia Primatologica. 2001;72(3):156. | Case report |
| Bottcher, D.; Bangoura, B.; Schmaschke, R.; Muller, K.; Fischer, S.; Vobis, V.; Meiler, H.; Wolf, G.; Koller, A.; Kramer, S.; Overhoff, M.; Gawlowska, S.; Schoon, H.-A. Diagnostics and epidemiology of alveolar echinococcosis in slaughtered pigs from large-scale husbandries in Germany. Parasitology research. 2013;112(2):629–36. | Case report |
| Boucher, J.M.; Hanosset, R.; Augot, D.; Bart, J.M.; Morand, M.; Piarroux, R.; Pozet-Bouhier, F.; Losson, B.; Cliquet, F. Detection of *Echinococcus multilocularis* in wild boars in France using PCR techniques against larval form. Veterinary Parasitology. 2005;129(3–4):259–66. | Case report |
| Boufana, B.; Craig, P.S.; Stidworthy, M.F.; Masters, N.; Greenwood, A.G.; Bell, S.; Wood, R.; Chantrey, J.; Unwin, S.; Lawrence, R.P.; Potter, A.; McGarry, J.; Redrobe, S.; Killick, R.; Wyatt, K.; Foster, A.P.; Mitchell, S.; Sako, Y.; Nakao, M.; Ito, A.; Lord, B. *Echinococcus* and *Taenia* spp. from captive mammals in the United Kingdom. Veterinary Parasitology. 2012;190(1–2):95–103. | Case report |
| Boussinesq, M.; Bresson, S.; Liance, M.; Houin, R. The muskrat as a new natural intermediate host for *E. multilocularis* in France. Bulletin de la Societe Francaise de Parasitologie. 1985;2:41. | Case report |
| Brack, M.; Tackmann, K.; Conraths, F.J.; Rensing, S. Alveolar hydatidosis (*Echinococcus multilocularis*) in a captive rhesus monkey (*Macaca mulatta*) in Germany. Tropical Medicine & International Health. 1997;2(8):754–9. | Case report |
| Cirovic D.; Pavlovic, I.; Kulisic, Z.; Ivetic, V.; Penezic, A.; Cosic, N. *Echinococcus multilocularis* in the European beaver (*Castor fibre* L.) from Serbia: first report. The Veterinary record. 2012;171(4):100. | Case report |
| Deblock, S.; Prost, C.; Walbaum, S.; Petavy, A.F. *Echinococcus multilocularis*: A rare cestode of the domestic cat in France. International Journal for Parasitology. 1989;19(6):687–8. | Case report |
| Eckert, J.; Muller, B.; Partridge, A.J. The domestic cat and dog as natural definitive hosts of *Echinococcus (alveococcus) multilocularis* in southern federal republic of Germany. Tropenmedizin und Parasitologie. 1974;25(3):334–7. | Case report |
| Geisel, O.; Barutzki, D.; Minkus, G.; Hermanns, W.; Loescher, T. Dogs as carriers (intermediate host) of larvae of *Echinococcus multilocularis*. Kleintierpraxis. 1990;35(6):275–80. | Case report |
| Genov, T.P.; Svilenov, D.K.; Polyakova-Krusteva, O.T. The natural occurrence of *Alveococcus multilocularis* in the *Microtus nivalis* in Bulgaria. Doklady Bolgarskoi Akademii Nauk (Comptes Rendus de l’Academie Bulgare des Sciences). 1980;33(7):981–4. | Case report |
| Karamon, J.; Sroka, J.; Cencek, T. The first detection of *Echinococcus multilocularis* in slaughtered pigs in Poland. Veterinary parasitology. 2012;185(2–4):327–9. | Case report |
| Matz-Rensing K.; Zoller M.; Habermalz, G.; Dinkel, A.; Kaup, F.J. Alveolar echinococcosis in a dog. Kleintierpraxis. 2002;47(11):683. | Case report |
| Meyer, A.; Conraths, F.J.; Schneemann, C.; Wienrich, V.; Kershaw, O.; Gruber, A.D. Lethal alveolar echinococcosis in a dog: clinical symptoms and pathology. Berliner Munchener Tierarztliche Wochenschrift. 2013;126(9–10):408–14. | Case report |
| Petavy, A.F.; Deblock, S.; Gilot, B. Detection of the larva of *Taenia multilocularis* in 2 voles (*Microtus arvalis* and *Clethrionomys glareolus*) in a focus of alveolar echinococcosis in the Massif Central (France). Comptes rendus de l’Academie des sciences. Serie III, Sciences de la vie. 1984;299(18):735–7. | Case report |
| Petavy, A.F.; Prost, C.; Gevrey, J.; Gilot, B.; Deblock, S. Natural occurrence of *Echinococcus multilocularis* Leuckart, 1863, in the domestic cat: first report from a peri-urban area in France. Comptes Rendus de l’Academie des Sciences, III (Sciences de la Vie). 1988;307(9):553–6. | Case report |
| Petavy, A.F.; Deblock, S.; Walbaum, S.The house mouse: a potential intermediate host for *Echinococcus multilocularis* in France. Transactions of the Royal Society of Tropical Medicine and Hygiene. 1990;84(4):571–2. | Case report |
| Peters, M.; Kilwinski, J.; Wohlsein, P.; Conraths, F.J. Alveolar echinococcosis in a captive red-necked wallaby (*Macropus rufogriseus*). Berliner Und Munchener Tierarztliche Wochenschrift. 2010;123(1–2):63–9. | Case report |
| Pezelet, C. Hepatic alveolar echinococcosis (*Echinococcus multilocularis*) in a dog. Pratique Medicale et Chirurgicale de l’Animal de Compagnie. 2013;48(1):21–6. | Case report |
| Pfister, T.; Schad, V.; Schelling, U.; Lucius, R.; Frank, W. Incomplete development of larval *Echinococcus multilocularis* (Cestoda: Taeniidae) in spontaneously infected wild boars. Parasitology research. 1993;79(7):617–8. | Case report |
| Rietschel, W.; Kimmig, P. Alveolar echinococcosis in a cynomolgus monkey. Tierarztliche Praxis. 1994;22(1):85–8. | Case report |
| Scharf, G.; Deplazes, P.; Kaser-Hotz, B.; Borer, L.; Hasler, A.; Haller, M.; Fluckiger, M. Radiographic, ultrasonographic, and computed tomographic appearance of alveolar echinococcosis in dogs. Veterinary radiology & ultrasound: the official journal of the American College of Veterinary Radiology and the International Veterinary Radiology Association. 2004;45(5):411–8. | Case report |
| Staebler, S.; Steinmetz, H.r; Keller, S.; Deplazes, P. First description of natural *Echinococcus multilocularis* infections in chinchilla (*Chinchilla laniger*) and Prevost’s squirrel (*Callosciurus prevostii borneoensis*). Parasitology research. 2007;101(6):1725–7. | Case report |
| Tackmann, K.; Beier, D. First case of *Echinococcus multilocularis* in foxes in north-west Brandenburg. Tieraerztliche Umsch. 1992;47(4):276 p. | Case report |
| Umhang, G; Lahore, J.; Nicolier, A.; Boue, F. *Echinococcus multilocularis* infection of a ring-tailed lemur (*Lemur catta*) and a nutria (*Myocastor coypus*) in a French zoo. Parasitology International. 2013;62(6):561–3. | Case report |
| van Riel, A.; Sjollema, B.; Klarenbeek, S.; van der Giessen, J. A dog with alveolar echinococcosis: the larval stage of the fox tapeworm. Tijdschrift voor diergeneeskunde. 2007;132(21):828–31. | Case report |
| Wohlsein, P.; Algermissen, D.; Wohlsein, P.; Grimm, F.; Grammell, T. Canine alveolar echinococcosis-a case report. Kleintierpraxis. 2009;54(10):558. | Case report |
| Worbes, H.; Schacht, K.H.; Eckert, J. *Echinococcus multilocularis* in a swamp beaver (*Myocaster coypus*). Angewandte Parasitologie. 1989;30(3):161–5. | Case report |
| Anonymous. UK meets *E multilocularis* surveillance requirements, says EFSA. The Veterinary record. 2013;173(20):486. | No epidemiological data |
| Antolova, D.; Miterpakova, M. Northern Slovakia-highly endemic area ofalveolar echinococcosis. Tropical Medicine and International Health. 2013;18(S1):223. | No epidemiological data |
| Carvalho-Varela, M. *Echinococcus multilocularis* in the Iberian Peninsula. Anais da Escola Superior de Medicina Veterinaria, Lisboa. 1986;23–24:102–17. | No epidemiological data |
| Dinkel, A.; Kern, S.; Brinker, A.; Oehme, R.; Vaniscotte, A.; Giraudoux, P.; Mackenstedt, U.; Romig, T. A real-time multiplex-nested PCR system for coprological diagnosis of *Echinococcus multilocularis* and host species. Parasitology Research. 2011;109(2):493–8. | No epidemiological data |
| Fisher, M. Update on *Echinococcus multilocularis* with particular emphasis on its impact on humans. The Veterinary Nurse. 2014;5(4):202–6. | No epidemiological data |
| Giraudoux, P.; Pleydell, D.; Raoul, F.; Vaniscotte, A.; Ito, A.; Craig, P.S. *Echinococcus multilocularis*: why are multidisciplinary and multiscale approaches essential in infectious disease ecology? Tropical Medicine and Health. 2007;35(4):293–9. | No epidemiological data |
| Graham, A.J.; Danson, F.M.; Craig, P.S. Ecological epidemiology : the role of landscape structure in the transmission risk of the fox tapeworm *Echinococcus multilocularis* (Leukart 1863) (Cestoda: Cyclophyllidea: Taeniidae). Progress in Physical Geography. 2005;29(1):77–91. | No epidemiological data |
| Hansen, F.; Tackmann, K.; Jeltsch, F.; Wissel, C.; Thulke, H.-H. Controlling *Echinococcus multilocularis* -ecological implications of field trials. Prev Vet Med. 2003;60(1):91–105. | No epidemiological data |
| Hegglin, D.; Deplazes, P. Control strategy for *Echinococcus multilocularis*. Emerging Infectious Diseases. 2008;14(10):1626–28. | No epidemiological data |
| Houin, R.; Liance, M.; Dumas, J.M.; Puel, F. *Arvicola terrestris* reservoir and focalizing element of alveolar echinococcosis in France. Molecular and Biochemical Parasitology. 1982;Suppl:258–9. | No epidemiological data |
| Kern, P.; Giraudoux, P.; Romig, T. Spatial and temporal epidemiology of *Echinococcus multilocularis*: Results of the European project echinorisk. American Journal of Tropical Medicine and Hygiene. 2005;73(6)S:28. | No epidemiological data |
| Keyserlingk, M.; Thoms, B.; Koerfer, K.H. Demonstration of the prevalence of *Echinococcus multilocularis* in foxes in Lower Saxony. Tieraerztliche Umsch. 1994;49(6):374. | No epidemiological data |
| Knapp, J.; Bart, J.M.; Glowatzki, M.L.; Ito, A.; Gerard, S.; Maillard, S.; Piarroux, R.; Gottstein, B. Assessment of use of microsatellite polymorphism analysis for improving spatial distribution tracking of *echinococcus multilocularis*. Journal of clinical microbiology. 2007;45(9):2943–50. | No epidemiological data |
| Knapp, J.; Bart, J.-M.; Giraudoux, P.; Glowatzki, M.-L.; Breyer, I.; Raoul, F.; Deplazes, P.; Duscher, G.; Martinek, K.; Dubinsky, P.; Guislain, M.-H.; Cliquet, F.; Romig, T.; Malczewski, A.; Gottstein, B.; Piarroux, R. Genetic diversity of the cestode *Echinococcus multilocularis* in red foxes at a continental scale in Europe. PLoS neglected tropical diseases. 2009;3(6):e452. | No epidemiological data |
| Knapp, J.; Bart, J.M.; Maillard, S.; Gottstein, B.; Piarroux, R. The genomic *Echinococcus* microsatellite EmsB sequences: from a molecular marker to the epidemiological tool. Parasitology. 2010;137(3):439–49. | No epidemiological data |
| Knapp, J.; Staebler, S.; Bart, J.M.; Stien, A.; Yoccoz, N.G.; Drogemuller, C.; Gottstein, B.; Deplazes, P. *Echinococcus multilocularis* in Svalbard, Norway: microsatellite genotyping to investigate the origin of a highly focal contamination. Infection, genetics and evolution: journal of molecular epidemiology and evolutionary genetics in infectious diseases. 2012;12(6):1270–4. | No epidemiological data |
| Konyaev, S.V.; Yanagida, T.; Nakao, M.; Ingovatova, G.M.; Shoykhet, Y.N.; Bondarev, A.Y.; Odnokurtsev, V.A.; Loskutova, K. S.; Lukmanova, G.I.; Dokuchaev, N.E.; Spiridonov, S.; Alshinecky, M.V.; Sivkova, T.N.; Andreyanov, O.N.; Abramov, S.A.; Krivopalov, A.V.; Karpenko, S. V.; Lopatina, N.V.; Dupal, T.A.; Sako, Y.; Ito, A. Genetic diversity of *Echinococcus* spp. in Russia. Special Issue: Control of cestode zoonoses in Asia: role of basic and applied science. Parasitology. 2013;140(13):1637–47. | No epidemiological data |
| Pfister, T.; Schad, V.; Frank, W. Serological studies on *Echinococcus multilocularis* in the definitive host. Mitteilungen der Oesterreichischen Gesellschaft fuer Tropenmedizin und Parasitologie. 1991;13:31–9. | No epidemiological data |
| Pleydell, D.R.J.; Raoul, F.; Tourneux, F.; Danson, F.M.; Graham, A.J.; Craig, P.S.; Giraudoux, P. Modelling the spatial distribution of *Echinococcus multilocularis* infection in foxes . Acta tropica. 2004;91(3):253–65. | No epidemiological data |
| Raoul, F.; Michela,t D.; Ordinaire, M.; Decote, Y.; Aubert, M.; Delattre, P.; Deplazes, P.; Giraudoux, P. *Echinococcus multilocularis*: secondary poisoning of fox population during a vole outbreak reduces environmental contamination in a high endemicity area. International Journal For Parasitology. 2003;33(9):945–54. | No epidemiological data |
| Robardet, E.; Caillot, C.; Augot, D.; Boue, F.; Barrat, J.; Giraudoux, P. Fox defecation behaviour in relation to spatial distribution of voles in an urbanised area: An increasing risk of transmission of *Echinococcus multilocularis*? International Journal for Parasitology. 2011;41(2):145–54. | No epidemiological data |
| Romig, T.; Thoma, D.; Weible, A.-K. *Echinococcus multilocularis* - A zoonosis of anthropogenic environments?. Journal of Helminthology. 2006;80(2):207–12. | No epidemiological data |
| Schantz, P.M. Editorial: The burden of echinococcosis . American Journal of Tropical Medicine and Hygiene. 2005;73(1):1. | No epidemiological data |
| Tackmann, K.; Goretzki, J.; Sutor, A.; Schwarz, S.; Poetzsch, C.; Conraths, F.J. Is the neozoan raccoon dog epidemiologically relevant as a definitive host of *Echinococcus multilocularis*? International Journal of Medical Microbiology. 2004;293(S38):58–59. | No epidemiological data |
| von Keyserlingk-Eberius, M. A fifteen-year-investigation on the prevalence of *Echinococcus multilocularis* in the red fox population of Lower Saxony - An overview. Journal Of Consumer Protection And Food Safety. 2008;3(4):421–8. | No epidemiological data |
| Gemmell, M.A. The fox as a definitive host of *Echinococcus* and its role in the spread of hydatid disease. Bulletin of the World Health Organisation. 1959;20(1):87–99. | Data on *E.granulosus* |
| Lis, H.; Gorski, K. The hydatid tapeworm - pig echinococossis. Zycie Weterynaryjne. 2012;87(3):241–2. | Data on *E.granulosus* |
| Loos-Frank, B.; Zeyhle, E. The intestinal helminths of the red fox and some other carnivores in southwest Germany. Zeitschrift fur Parasitenkunde. 1982;67(1):99–113. | Data on *E.granulosus* |
| Lorenzini, R.; Ruggieri, A. Distribution of echinococcosis/hydatidosis in Italy. Journal of Helminthology. 1987;61(3):261–7. | Data on *E.granulosus* |
| Matossian R.M.; Rickard M.D.; Smyth J.D. Hydatidosis: a global problem of increasing importance. Bulletin of the World Health Organisation. 1977;55(4):499–507. | Data on *E.granulosus* |
| Seimenis, A. Overview of the epidemiological situation on echinococcosis in the Mediterranean region. Acta tropica. 2003;85(2):191–5. | Data on *E.granulosus* |
| Varcasia, A.; Tanda, B.; Giobbe, M.; Solinas, C.; Pipia, A.P.; Malgor, R.; Carmona, C.; Garippa, G.; Scala, A. Cystic echinococcosis in Sardinia: farmers’ knowledge and dog infection in sheep farms. Veterinary parasitology. 2011;181(2–4):335–40. | Data on *E.granulosus* |
| Anonymous. Alveolar echinococcosis. Weekly epidemiological record /Health Section of the Secretariat of the League of Nations. 1990;65(6):37–8. | Data on human |
| Craig, P.S. Current research in echinococcosis. Parasitology Today. 1994;10(6):209–11. | Data on human |
| Craig, P.S.; Rogan, M.T.; Allan, J.C. Detection, screening and community epidemiology of taeniid cestode zoonoses: cystic echinococcosis, alveolar echinococcosis and neurocysticercosis. Advances in parasitology. 1996;38:169–250. | Data on human |
| Fesseler, M.; Muller, B.; Eckert, J. Comparison of the geographic distribution and prevalence of *Echinococcus multilocularis* and rabies infection. Tierarztliche Umschau. 1991;46(5):287–92. | Data on human |
| Roy, M.; Morin, B.; Petavy, A. G.; Cambon, M.; Baril, A. First observations of the alveolar echinococcosis in the Auvergne. Nouvelle Presse Medicale. 1977;6(43):4070–71. | Data on human |
| Zeyhle, E. Distribution of *Echinococcus multilocularis* in south-western Germany. Probleme der Echinokokkose unter Berucksichtigung parasitologischer und klinischer Aspekte. (Aktuelle Probleme in Chirurgie und Orthopadie, Band 23). 1982:26–32. | Data on human |
| Bregadze, I.L.; Semenov, V.S. Nosogeography of the alveolar form of echinococcosis in Siberia. Med. Parazit. I Parazit. Bol. 1961;30(2):168–72. | Not included languages |
| Lukashenko, N.P.; Zorikhina, V.I. The aetiology of alveolar echinococcosis in the central districts of the barabinsk forest-steppe (novosibirsk region). Med. Parazit. I Parazit. Bol. 1961;30(2):159–68. | Not included languages |
| Masur, O.E.; Fomina, A.S. Revelation of *Echinococcus multilocularis* (Leuckart, 1856) in the muskrat (*Ondatra zibethicus*) of the delta of the Selenga River (Russia). Russian Journal of Biological Invasions. 2012; 3(4):251–4. | Not included languages |
| Petrovic, Z. Taeniasis, cystocercosis, echinococcosis and hydatidosis in Yugoslavia at the present time. Parasitologia Hungarica. 1979;12:37–9. | Not included languages |
| Al-Sabi, M.N.S.; Enemark, H.L.; Chriel, M.; Jensen, T.H. Updates on the surveillance program on parasites of raccoon dogs and foxes in Denmark 2011–2012. Tropical Medicine and International Health. 2013;18(S1):96. | Duplicated |
| Enemark, H.L.; Al-Sabi, M.N.; Knapp, J.; Staahl, M.; Chriel, M. Detection of a high-endemic focus of *Echinococcus multilocularis* in red foxes in southern Denmark, January 2013. Eurosurveillance: European communicable disease bulletin. 2013;18(10):20420pp. | Duplicated |
| Staubach, C.; Hoffmann, L.; Schmid, V.; Ziller, M.; Tackmann, K.; Conraths, F.J. Echinococcosis surveillance: bayesian time-space analysis of *Echinococcus multilocularis* infections in foxes in Thuringia, Germany. Epidemiologie et Sante Animale. 2011;59-60:23–5. | Duplicated |
| Vergles Rataj, A.V.; Bidovec, A.; Zele, D.; Vengust G. *Echinococcus multilocularis* in the red fox (*Vulpes vulpes*) in Slovenia. European Journal of Wildlife Research. 2010;56:819–22. | Duplicated |
