# Supplementary material for: The geographical distribution and prevalence of Echinococcus multilocularis in animals in the European Union and adjacent countries: a systematic review and meta-analysis
Source: Parasit Vectors. 2016 Sep 28;9:519. doi: 10.1186/s13071-016-1746-4 (PMC5039905; doi:10.1186/s13071-016-1746-4)
Supplement: Additional file 5: Table S1. — Sampling strategy for red foxes (and Arctic foxes in Svalbard Islands, Norway). (DOC 66 kb) [file 13071_2016_1746_MOESM5_ESM.doc]

***S1 Table. Sampling strategy for red foxes (and Arctic foxes in Svalbard Islands, Norway).***

| Country | Sampling strategy | | | |
| --- | --- | --- | --- | --- |
|  | N^o^ papers rabies program | N ^o^ papers hunted or shot | N ^o^ papers convenience sampling or control programmes | N ^o^ papers with data not reported |
| Austria | 1 | 4 | 2 | 1 |
| Belgium | 0 | 9 | 1 | 2 |
| Switzerland | 3 | 11 | 2 | 2 |
| Czech Republic | 2 | 2 | 2 | 4 |
| Germany | 3 | 21 | 15 | 11 |
| Denmark | 0 | 3 | 1 | 0 |
| Estonia | 0 | 3 | 1 | 0 |
| Spain | 0 | 0 | 1 | 0 |
| Finland | 1 | 1 | 2 | 1 |
| France | 2 | 13 | 2 | 4 |
| Croatia | 1 | 2 | 3 | 3 |
| Hungary | 1 | 3 | 1 | 0 |
| Ireland | 0 | 1 | 2 | 1 |
| Italy | 0 | 8 | 1 | 0 |
| Lithuania | 0 | 1 | 0 | 0 |
| Luxemburg | 2 | 0 | 2 | 0 |
| Latvia | 0 | 2 | 0 | 0 |
| Netherlands | 2 | 7 | 1 | 1 |
| Norway | 0 | 3 | 2 | 0 |
| Norway (Arctic fox) | 0 | 1 | 0 | 1 |
| Poland | 0 | 16 | 0 | 1 |
| Romania | 0 | 3 | 0 | 0 |
| Sweden | 1 | 4 | 2 | 1 |
| Slovenia | 0 | 2 | 0 | 0 |
| Slovakia | 1 | 9 | 4 | 4 |
| United Kingdom | 0 | 2 | 3 | 1 |
| Ukraine | 0 | 2 | 0 | 0 |
| Total (241) | 20 | 133 | 50 | 38 |
